# The genome variation and developmental transcriptome maps reveal genetic differentiation of skeletal muscle in pigs

**DOI:** 10.1371/journal.pgen.1009910

**Published:** 2021-11-15

**Authors:** Yalan Yang, Junyu Yan, Xinhao Fan, Jiaxing Chen, Zishuai Wang, Xiaoqin Liu, Guoqiang Yi, Yuwen Liu, Yongchao Niu, Longchao Zhang, Lixian Wang, ShuaiCheng Li, Kui Li, Zhonglin Tang

**Affiliations:** 1 Shenzhen Branch, Guangdong Laboratory for Lingnan Modern Agriculture, Agricultural Genomics Institute at Shenzhen, Chinese Academy of Agricultural Sciences, Shenzhen, China; 2 Genome Analysis Laboratory of the Ministry of Agriculture and Rural Affairs, Agricultural Genomics Institute at Shenzhen, Chinese Academy of Agricultural Sciences, Shenzhen, China; 3 Kunpeng Institute of Modern Agriculture at Foshan, Foshan, China; 4 Department of Computer Science, City University of Hong Kong, Kowloon, Hong Kong, China; 5 Guangxi Engineering Centre for Resource Development of Bama Xiang Pig, Bama, China; 6 Biozeron Shenzhen, Inc., Shenzhen, China; 7 Institute of Animal Sciences, Chinese Academy of Agricultural Sciences, Beijing, China; 8 Research Centre of Animal Nutritional Genomics, State Key Laboratory of Animal Nutrition, Institute of Animal Sciences, Chinese Academy of Agricultural Sciences, Shenzhen, China; Uppsala University, SWEDEN

## Abstract

Natural and artificial directional selections have resulted in significantly genetic and phenotypic differences across breeds in domestic animals. However, the molecular regulation of skeletal muscle diversity remains largely unknown. Here, we conducted transcriptome profiling of skeletal muscle across 27 time points, and performed whole-genome re-sequencing in Landrace (lean-type) and Tongcheng (obese-type) pigs. The transcription activity decreased with development, and the high-resolution transcriptome precisely captured the characterizations of skeletal muscle with distinct biological events in four developmental phases: Embryonic, Fetal, Neonatal, and Adult. A divergence in the developmental timing and asynchronous development between the two breeds was observed; Landrace showed a developmental lag and stronger abilities of myoblast proliferation and cell migration, whereas Tongcheng had higher ATP synthase activity in postnatal periods. The miR-24-3p driven network targeting insulin signaling pathway regulated glucose metabolism. Notably, integrated analysis suggested *SATB2* and *XLOC_036765* contributed to skeletal muscle diversity via regulating the myoblast migration and proliferation, respectively. Overall, our results provide insights into the molecular regulation of skeletal muscle development and diversity in mammals.

## Author summary

Compared with the commercial breeds, Chinese local pig breeds have lower growth rates, higher fat content and better meat quality, because they have adapted to local environment and have not been strongly selected. These differences make them as exceptional resources to identify candidate markers for the improvement of meat production traits in pig breeding. In this study, we compared the genome and skeletal muscle transcriptome differences between Chinese local Tongcheng and commercial Landrace pigs, and uncovered the genetic regulation of coding and non-coding RNAs, such as *SATB2* and *XLOC_036765*, in skeletal muscle development and diversity. This study enhances our understanding of the genetic basis of skeletal muscle development and diversity, and provide useful molecular markers for the genetic improvement of meat production traits.

## Introduction

Skeletal muscle development is a complex and dynamically regulated process of temporally separated but highly coordinated events including the determination of myogenic progenitor cells, myoblast proliferation and differentiation, myoblast fusion, and muscle growth and maturation [1]. The development of skeletal muscle in mammals is biphasic and involves the primary generation of myotubes formed during embryonic development and a secondary wave of myogenesis occurring around midgestation, thus establishing the total number of myofibers [2,3] and greatly determining the capacity for postnatal muscle fiber growth and hypertrophy [4,5]. After birth, cell proliferation decreases but muscle grows and adapts, largely by remodeling pre-existing fibers [6]. Meanwhile, a transition of slow-oxidative to fast-glycolytic fiber types happens during the neonatal period. All these events are precisely controlled by complex networks of spatially and temporally expressed genes according to strict time-sequence expression [7,8], including the Pax family (*Pax3* and *Pax7*), the myogenic regulatory factor (*MRF*) family, and the myocyte enhancer factor 2 (*MEF2*) family [9].

The domestic pig (*Sus scrofa* domestica) is not only a main protein source for humans, but also is an important biomedical model animal [10–12]. The effects of geographical divergence, local adaptation, and artificial selection result in significantly genetic and phenotypic differentiation between pig breeds [13,14]. Commercial Western breeds, such as Landrace pigs, have been intensively selected for rapid, large, and efficient accretion of muscle, while Chinese indigenous breeds, such as Tongcheng pigs, have lower growth rates, higher fat content and better meat quality [15]. These differences make them as an exceptional model to elucidate the underlying mechanisms of phenotype differentiation within species, especially in muscle mass and quality [15–17]. The muscle mass and quality mainly depend on the development of skeletal muscle. Although recent studies have accumulated knowledge of skeletal muscle development in pigs [15–17]. However, the genetic basis of transcriptome dynamic and diversity in skeletal muscle development remain unclear.

To further understand the genetic regulation of dynamic and diversity of skeletal muscle development, here, we profiled dynamic RNA-seq and miRNA-seq of skeletal muscle across 27 developmental time points (including 15 prenatal and 12 postnatal time points) in Landrace (lean-type Western breed) and Tongcheng (obese-type Chinese local breed) pigs. We analyzed the molecular characteristics, gene co-expression and interaction network of skeletal muscle development, and compared the similarity and difference between Landrace and Tongcheng pigs. The integrative analysis of transcriptome and genome revealed the genetic regulation of coding and non-coding RNAs, such as *SATB2* and *XLOC_036765*, in skeletal muscle development and diversity *in vivo* and *in vitro*. This study not only provides a valuable resource for animal breeding, but also is helpful for understanding the skeletal muscle biology and relevant diseases.

## Results

### Overview the transcriptome of developing *longissimus dorsi*

**Fig 1:**
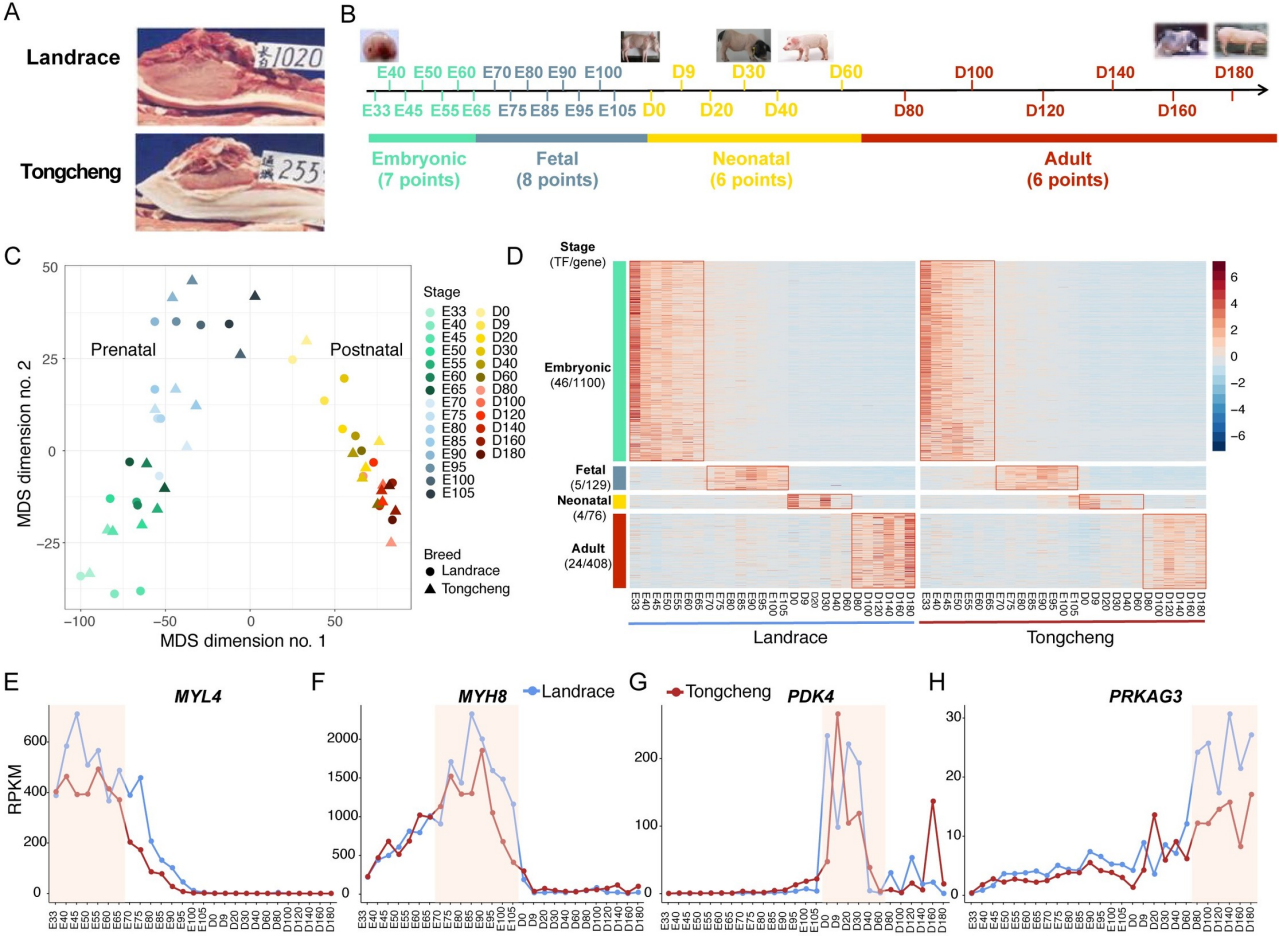
Global view of the pig skeletal muscle transcriptome. (**A**) The cross section of *longissimus dorsi* and subcutaneous fat between the 6th and 7th ribs in 10-month-old Landrace and Tongcheng pigs. (**B**) Skeletal muscle sample collections from Landrace and Tongcheng pigs at 27 developmental time points (E33 to D180) collected for RNA-seq and miRNA-seq analysis. E, embryonic days, D, postnatal days. (**C**) Multidimensional scaling (MDS) plot showing similarities and differences in genome-wide expression profiles among skeletal muscle samples from Landrace and Tongcheng pigs. MDS was performed on data from all expressed genes to represent each sample as a single point colored by the developmental period. The Euclidean distance of log2-transformed RPKM values was used to measure pairwise similarity. (**D**) Relative expression pattern of phase-specific expressed genes in the embryonic, fetal, neonatal, and adult periods. (**E**) The expression of representative phase-specific genes (*MYL4*, *MYH8*, *PDK4*, and *PRKAG3*) during skeletal muscle development.

Landrace and Tongcheng pigs have significant phenotype differences in muscle mass, growth rate, lean percentage, and meat quality (Figs 1A and S1). To understand the molecular regulation of transcriptome dynamic and diversity in skeletal muscle development, we profiled the transcriptome and miRNAome of skeletal muscle (*longissimus dorsi*) across 27 developmental time points (from embryonic day 33 to postnatal day 180) in Landrace and Tongcheng pigs (Fig 1B). We obtained 4.3 billion 90-nt pair-end reads for RNA-Seq (S1 Table) and 776 million 49-nt single-end reads for miRNA-seq analyses (S2 Table). Using strict criteria for long noncoding RNA (lncRNA) identification as described previously [18,19], we identified a total of 1,661 novel long intergenic noncoding RNA (lincRNAs), corresponding to 3,269 transcripts in skeletal muscle (S3 Table). The characteristics of novel lincRNAs agreed with our previous studies (S2 Fig) [18,19]. An average of 13,359 protein coding genes (PCGs, ranging from 12,138 to 14,141), 911 lincRNAs (ranging from 535 to 1,195) and 365 miRNAs (ranging from 292 to 422) was expressed (RPKM or TPM > 0.1) in analyzed skeletal muscles (S3 Fig).

We observed an obvious decrease in number of expressed genes and average abundances during skeletal muscle development (S3A–S3D Fig), consistent with the decrease expression of cell cycle marker *MKI67* and myoblast proliferation regulator *MYF5* during development (S3E Fig). These results suggested that skeletal muscle had higher transcriptional activity at prenatal stages than at postnatal ones. In addition, 66.62% (10,825/16,249) of genes (PCGs and lincRNAs) had high coefficients of variation (CV > 0.5, S3F Fig), indicating that the majority of genes were dynamically expressed during skeletal muscle development.

Next, multidimensional scaling (MDS) analysis was performed to explore the variance of developing skeletal muscle at transcriptional level. We found that the skeletal muscle samples were ordered by developmental stages in a characteristic inverted U shape (Fig 1C). The first dimension obviously separated the prenatal from the postnatal samples, whereas the second dimension appeared to align with development within both the prenatal and postnatal muscles, suggesting effective reconstruction of the transcriptional dynamics of skeletal muscle development (Fig 1C). Based on the MDS analysis and the characteristics of development, the skeletal muscles at 27 time points were obviously divided into 4 developmental phases: Embryonic (E33 to E65, represented primary myotube formation), Fetal (E70 to E105, represented secondary myotube formation), Neonatal (D0 to D60, represented myofiber type transformation) and Adult (D80 to D180, represented skeletal muscle growth and maturation) periods (Fig 1B).

### Phase-specific characteristics during skeletal muscle development

To explore the molecular characteristic of skeletal muscle at different developmental phases, we first examined differentially expressed genes (DEGs) between adjacent phases (S4A–S4C Fig and S4 Table). The results revealed that more DEGs (5,642) were detected in the fetal-neonatal groups than in other comparisons, indicating that gene expression in skeletal muscle changed significantly after birth. Compared with the embryonic period, the genes involved in the muscle system process and muscle contraction were up-regulated, while the genes associated with nervous system development were down-regulated at the fetal period (S4D Fig). Compared with the fetal period, the up-regulated genes at the neonatal period were significantly involved in oxidation-reduction, cellular respiration, and precursor metabolite and energy generation (S4E Fig). Compared to the neonatal period, genes related to the neurological system process were depressed in the adult skeletal muscles (S4F Fig).

Then, the phase-specific genes that pertained exclusively to the four phases of skeletal muscle development were identified. We defined the genes with significantly higher expression at a given phase than at the other three phases as phase-specific genes (FDR ≤ 0.05 and |fold change| ≥ 2). A considerable number of genes (1,100) with developmental phase-specific expression patterns were detected at the embryonic period, while 129, 76 and 408 genes were predominantly expressed in fetal, neonatal, and adult muscles (Fig 1D and S5 Table). It is noted that many well-known temporal-specific genes are involved in skeletal muscle development. For instance, myosin light chain 4 (*MYL4*) and myosin heavy chain 8 (*MYH8*) were specifically expressed at the embryonic and fetal period, respectively (Fig 1E and 1F). Pyruvate dehydrogenase kinase 4 (*PDK4*), which is critical for skeletal muscle metabolism [20], was specifically expressed at the neonatal stage (Fig 1G). Protein kinase AMP-activated non-catalytic subunit gamma 3 (*PRKAG3*) was overrepresented in adult muscles (Fig 1H). These results suggested that temporal-specific gene expression acts in concert with skeletal muscle development progress.

### Gene co-expression analysis during skeletal muscle development

**Fig 2:**
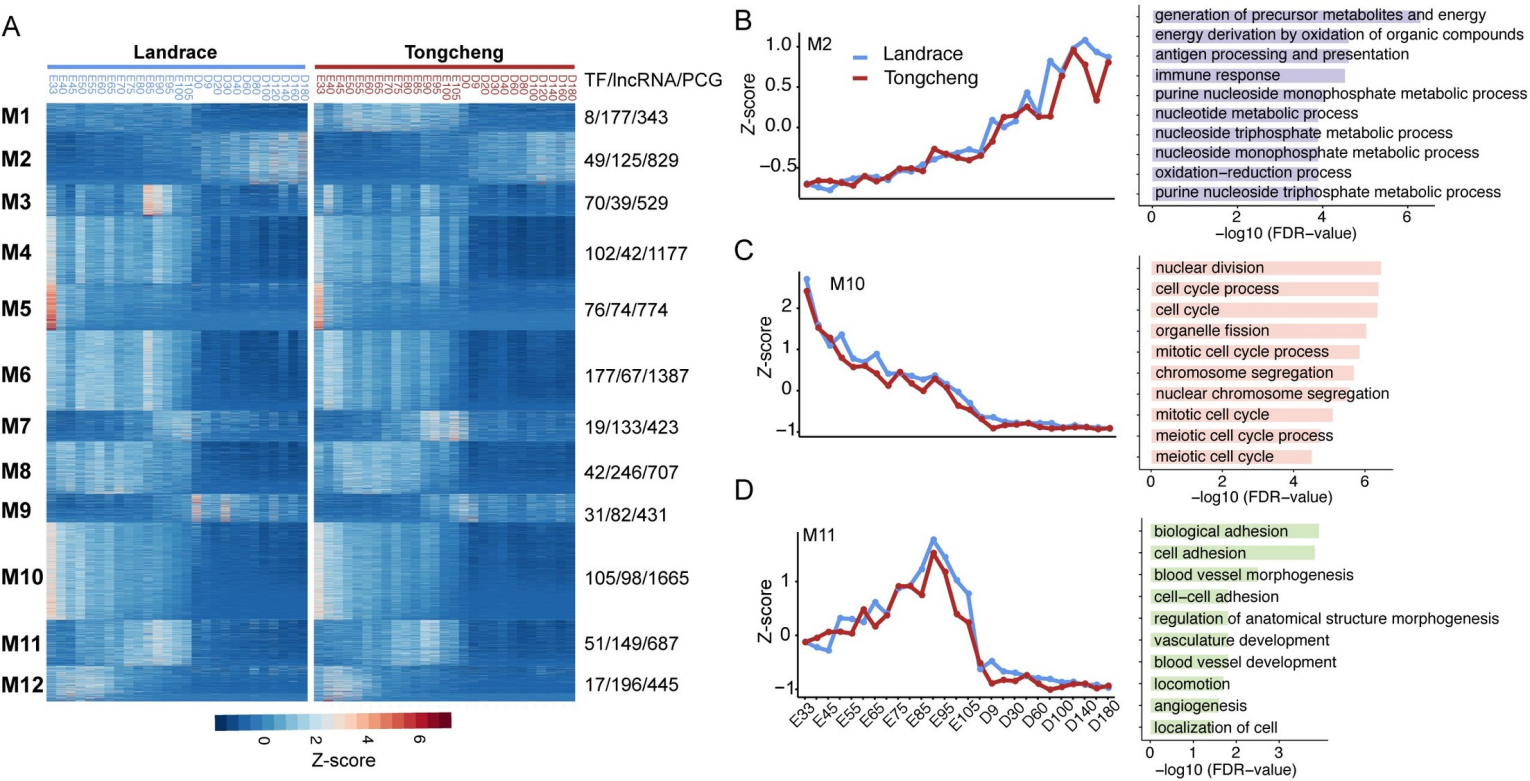
Gene co-expression network analyses during skeletal muscle development. (**A**) Heatmap showing the expression patterns of genes in the 12 co-expression modules. The number of TFs, lncRNAs and genes and in each module are shown on the right. (**B**) Module 2 (M2), *Left*, the median Z score of genes in M2 across skeletal muscle development, *Right*, the top 10 typical GO biological process terms associated with M2. (**C, D**) Same analyses as in **B**, but for M10 (**C**) and M11 (**D**).

To further provide insights into the functional transitions along skeletal muscle development, we carried out gene co-expression analysis using the k-means clustering algorithm. These genes with high variance (CV > 0.5) were divided into 12 co-expression modules (Fig 2A and S6 Table), which showed distinct temporal or stage-specific patterns with different biological functions. Among the 12 modules, module 2 (M2) consisted of 829 PCGs, 49 TFs and 125 lincRNAs with up-regulated patterns across development and highly expressed at the adult period. The PCGs in M2 were significantly enriched in generation of precursor metabolites and energy, oxidation-reduction process, and insulin signaling pathway (Fig 2B), including transcription factors *MRF4* [9], *YBX3* [21,22] and *TEAD1* [23], which have functional implications in the development of skeletal muscle. Muscle-specific regulatory factor 4 (*MRF4*, also known as *MYF6*) mainly drives the induction of terminal differentiation [24]. *YBX3* (also known as *CSDA*) was the most highly expressed TF in postnatal muscle. The negative expression correlation between *YBX3* and *MYOG* (Pearson *R* = -0.83, *P* = 6.35e-15, S5 Fig) coincides with previous reports in myogenesis [21,22]. By contrast, the M10 consisted of 1,665 PCGs, 105 TFs and 98 lincRNAs with gradually down-regulated expression during skeletal muscle development (Fig 2C), including myogenic regulatory factor *MYF5* [25], *TEAD2* [26] and *HMGB2* [27], and four insulin-like growth factor binding proteins (*IGFBP4*, *IGFBP5*, *IGF2BP2*, and *IGF2BP3*) (S6 Fig) [8,28], which played critical roles in myogenesis proliferation and differentiation, and muscle regeneration. The genes in M10 were enriched in GO terms for cell cycle associated processes (Fig 2C). The genes in M5 were specifically abundantly expressed at the early embryonic period, such as *SFRP1* [29], *FGFR2* [30], *BMP7* [31], *IGF2BP1* and *IGFBP2*, and significantly involved in nervous system development associated processes (S7A Fig). The genes in M11 were highly expressed at the fetal period, such as *EGFL7* and *HDAC7*. They were functionally related to biological adhesion, vasculature development, circulatory system development, blood vessel morphogenesis, and locomotion (Fig 2D). Additionally, the genes in M8 were highly expressed at the prenatal stage and were lowly expressed after birth, and functionally related to cell proliferation regulation, lipid transport and tissue remodeling, including 20 zf-C2H2 family members, such as *PLAGL1* and *ZNF283*, reflecting high proliferation activity of skeletal muscle at the prenatal stage (S7B Fig). The genes in M9 were highly expressed at the neonatal period, such as *PDHA1* and *COX1*, and functionally enriched in GO terms related to processes required for mature muscle cell activities such as cellular respiration and oxidation-reduction process (S7C Fig).

## ceRNA network during skeletal muscle development

Previous studies indicated that lncRNAs played an important role in the regulation of gene expression by acting as competing endogenous RNAs (ceRNAs) [32,33]. Based on predictions of binding sites and expression relationships among miRNAs, lincRNAs and mRNAs (S8A Fig), we compiled a comprehensive lincRNA-associated competing triplets cross-talk network of miRNA-mediated interactions to further understand the regulation of non-coding RNAs (miRNAs and lincRNA) in skeletal muscle development. By integrating 455 lincRNA-miRNA pairs with 4,727 miRNA-mRNA interactions, we identified 69,503 lincRNA-miRNA-mRNA interactions according to the common miRNA target sites for both lincRNA and mRNAs (S7 Table). These interactions formed two ceRNA networks, which exhibited opposing expression patterns during skeletal muscle development.

**Fig 3:**
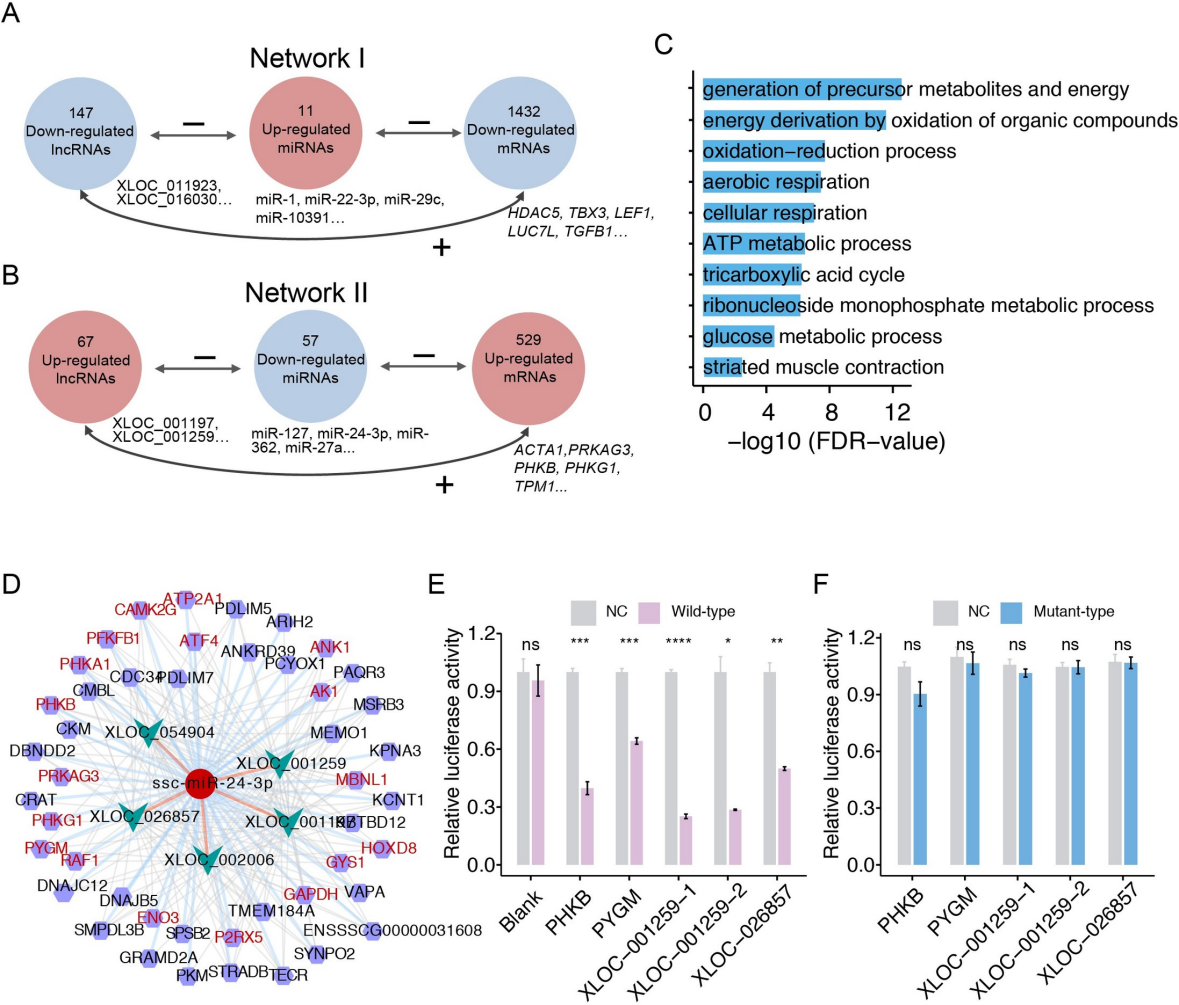
LincRNA-miRNA-mRNA regulatory network during skeletal muscle development. (**A, B**) Schematic diagram representing the lincRNAs-miRNA-mRNA axes that satisfied our criteria. “-” represents the negative correlation between the expression of lincRNAs/mRNAs and miRNAs. “+” represents the positive correlation between the expression of lincRNAs and mRNAs. The correlations are performed by Pearson’s correlation tests. (**C**) GO enrichment analyses of the mRNAs in network I. (**D**) Cytoscape showing the miR-24-3p involved lincRNA-miRNA-mRNA interactions, five lincRNAs and 40 mRNAs are targeted by miR-24-3p in this sub-network. The ellispe (dark red), arrow (turquoise), and hexagon (purple) nodes represent miR-24-3p, lincRNAs and mRNAs, respectively. mRNAs that are known to be involved in insulin signaling pathway are marked in red. The dark orange, light sky blue and grey edges represent the interactions between miR-24-3p and lncRNAs, between miR-24-3p and mRNAs, and between lincRNAs and mRNAs, respectively. (**E, F**) Validation of the targets of miR-24-3p by dual-luciferase reporter assay. Pig SkMCs were co-transfected with luciferase reporters carrying the wild-type (**E**) or mutated 3′-UTR (**F**) of four target genes (*PHGB*, *PYGM*, *XLOC-001259*, and *XLOC-026857*), as well as their corresponding miR-24-3p mimic/negative control duplexes. The data are represented as mean ± S.E.M (n = 3). *P < 0.01. **P < 0.01. ***P < 0.001. ****P < 0.0001. ns, no significant.

In network I, lincRNAs and mRNAs were down-regulated and miRNAs were up-regulated during skeletal muscle development (Figs 3A and S8B). This network included 64,359 interaction pairs comprising 147 lincRNAs, 1,432 mRNAs, and 11 miRNAs. Many of mRNAs in this network were associated with metabolic process and RNA splicing. The muscle-specific miR-1 [34] was up-regulated during development and potentially targeted three lincRNAs and 23 mRNAs, including *DDX5*, *TCF7L2* and *STIM1*, which have been reported involved in muscle cell differentiation. LincRNAs and mRNAs in network II were up-regulated during skeletal muscle development. This network included 5,144 interaction pairs comprised of 68 lincRNAs, 529 mRNAs and 57 miRNAs (Figs 3B and S8B). Functionally, these mRNAs were significantly associated with generation of precursor metabolites and energy, glucose metabolic process, muscle contraction, myofibril assembly, and oxidation reduction (Fig 3C), reflecting the potential functions of miRNAs and lincRNAs in postnatal skeletal muscle development via a ceRNAs mechanism. It is noteworthy that miR-24-3p, a negative regulator in insulin signaling pathway [35], could target five lincRNAs and 40 mRNAs in the network II (Fig 3D). miR-24-3p was mainly expressed at the prenatal skeletal muscle, and was higher expressed in Tongcheng than in Landrace pigs (S9 Fig). Interestingly, most of the 40 mRNAs were closely associated with glucose metabolic process and insulin signaling pathway. Dual-luciferase reporter gene assays were used to validate the ceRNAs for miR-24-3p in porcine skeletal muscle satellite cells (SkMCs), we found that the miR-24-3p minics significantly reduce the luciferase intensity of *PHKB*, *PYGM*, *XLOC-001259* and *XLOC-02685* (Fig 3E), while did not affect luciferase activity of the reporters with mutated binding sites (Fig 3F). These results indicated that miR-24-3p-mediated ceRNA network targeted insulin signaling pathway and contributed to glucose metabolism in skeletal muscle development.

### Comparative analysis between Landrace and Tongcheng pigs during skeletal muscle development

**Fig 4:**
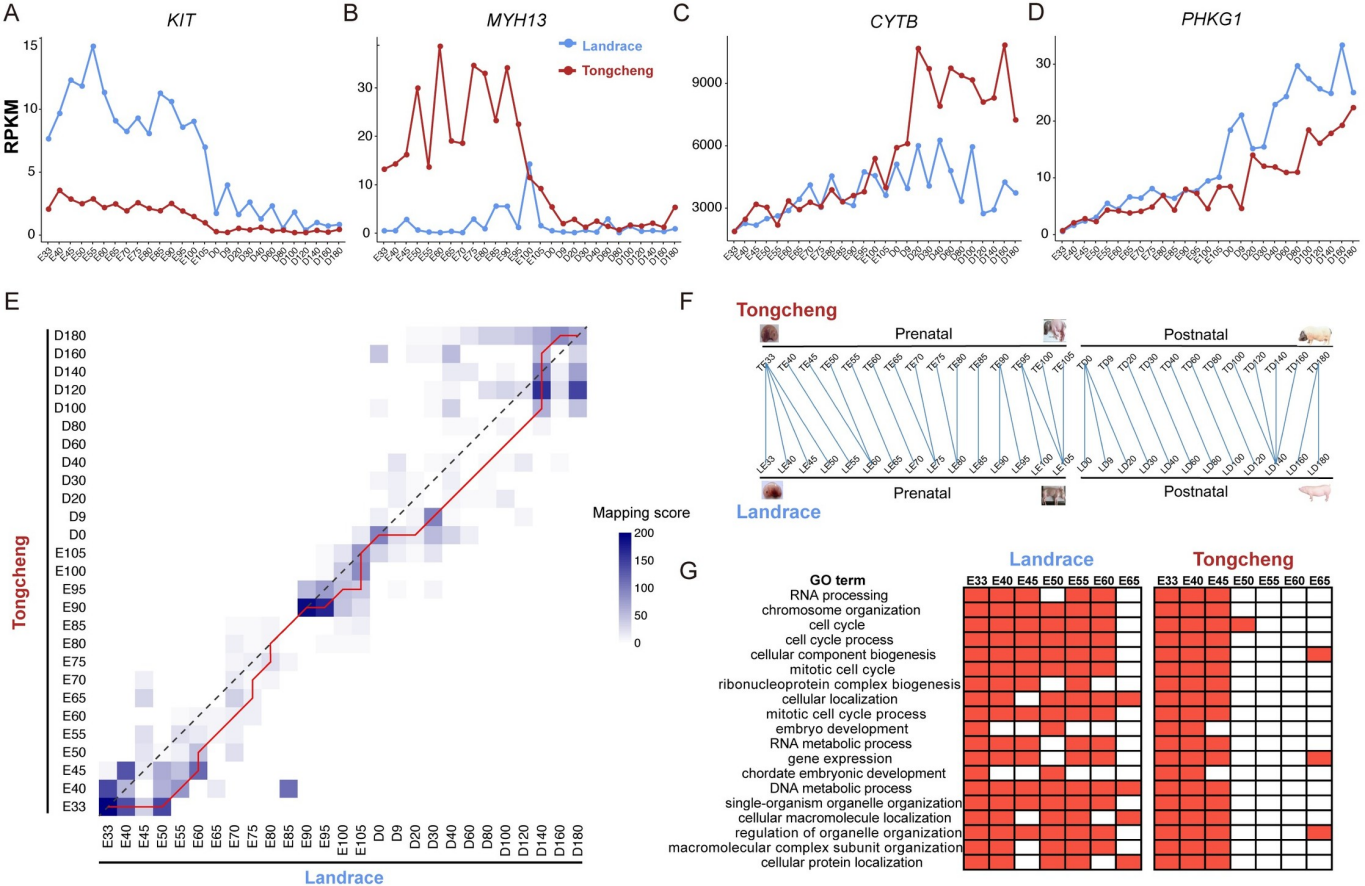
Comparison analysis between Landrace and Tongcheng pigs during skeletal muscle development. (**A-D**) The expression of representative differentially expressed genes (*KIT* (**A**), *MYH13* (**B**), *CYTB* (**C**), and *PHKG1* (**D**)) between Landrace and Tongcheng pigs during skeletal muscle development. (**E**) Comparison of transcriptome similarities across skeletal muscle development between Tongcheng and Landrace pigs. Mapping scores shown in the heatmap are–log10 Bonferroni-corrected *P*-values, calculated using the hypergeometric test. The red stair-step line was found by maximizing the sum of the mapping scores of the time point pairs it passes through to emphasize the two parallel collinear mapping patterns observed in Landrace and Tongcheng pigs at 27 developmental time points. (**F**) Summary of transcriptome comparisons between Tongcheng and Landrace pigs. (**G**) Comparison of GO enrichment analysis for development-associated genes (DAGs) during the embryonic period. A red block indicates that the GO term at this time point is significantly enriched (*P* < 0.05), a white block indicates no significance (*P* > 0.05).

Increases in muscle mass are mainly caused by an accumulation of synthesized protein and enlargement of the myofiber area during postnatal development through a process of hypertrophy [7]. The difference in muscle growth between Tongcheng and Landrace pigs is closely related to the synthesis and regulation of muscle protein at prenatal stages [15]. To understand the difference in muscle mass between lean-type and obese-type pigs, we first identified differentially expressed genes (DEGs) between Landrace and Tongcheng pigs at each phase, termed Type 1 DEGs (S8 Table). The result suggested that the genes related to cell migration were significantly higher expressed in Landrace at adult period (S9 Table), indicating a higher activation of cell migration and motility for Landrace compared with Tongcheng pigs. Subsequently, we performed multiple linear regression analysis based on the interaction between breeds and developmental stages (FDR ≤ 0.05) and identified 290 genes showing different expression trajectories across skeletal muscle development between the two breeds (Type 2 DEGs, S10 Table). GO analysis revealed that these genes were closely related to ATP synthesis, phosphorylation, and oxidation reduction (S10 Fig). The KIT proto-oncogene, receptor tyrosine kinase (*KIT*) gene was specifically highly expressed at prenatal periods of Landrace pigs (Fig 4A), which is likely caused by the dominant white mutation in Landrace pigs [36]. As expected, many Type 2 DEGs were associated with muscle system process, such as myosin heavy chain 13 (*MYH13*) (Fig 4B), which is responsible for the muscle’s high-speed performance. The higher expression of *MYH13* in Tongcheng pigs at the prenatal period might contribute to the differentiation of muscle fiber-type composition between these two pigs. Meanwhile, compared with the Landrace, the genes related to ATP synthesis coupled electron transport were up-regulated in Tongcheng, including NADH dehydrogenase, and mitochondrial and ribosomal genes, such as *CYTB* (Fig 4C). Interestingly, the mitochondrial biogenesis genes were more expressed in Tongcheng than in Landrace after birth (S11 Fig). The genes involved in glucose metabolism (*PRKAG3*, *PHKG1* and *PHKA1*) exhibited phase-specific expression patterns at the adult period (Fig 4D) and were targeted by miRNA-24-3p (Fig 3E). This fact revealed that the miR-24-3p/insulin signaling pathway axis contributed to the difference in glucose metabolism between Landrace and Tongcheng pigs.

Our high-temporal-resolution transcriptome also provided a way to precisely compare the similarities and differences of skeletal muscle development between Landrace and Tongcheng pigs. To this end, we used an approach described in a previous study to define development-associated genes (DAGs) as those that were highly expressed at a given time point relative to other time points throughout development [37]. The number of DAGs ranged from 95 to 6,423 during skeletal muscle development (S12 Fig). Notably, more DAGs were detected at the embryonic period and fewer DAGs were observed at the neonatal period. This phenomenon was consistent with observations of the number of phase-specific genes at these two periods. We next investigated the developmental similarities and differences between the two breeds by both statistically checking the dependence of the DAGs and testing the significance of the number of common DAGs. A red line (maximum trace) emphasized the parallel collinear mapping pattern, and many off-diagonal mappings matched at the same time points between the two breeds, especially at the embryonic and neonatal periods. A summary of developmental time point mapping between the two breeds was shown in Fig 4E. We found that Landrace and Tongcheng pigs mapped to each other at only eight time points (E33, E80, E85, E90, E105, D0, D140, and D180). At the other time points, however, Landrace hit Tongcheng earlier during development. This fact indicated that the development of skeletal muscle at those stages in Landrace obviously lagged compared to Tongcheng pigs.

As shown in Fig 4F, the E33 in Togncheng mapped to four successive time points (E33, E40, E45, and E50) in Landrace pigs. As expected, many DAGs at the embryonic period closely associated with the cell cycle. Interestingly, the DAGs in Landrace were temporally activated in the whole embryonic period (from E33 to E60), however, were repressed after E45 in Tongcheng pigs (Fig 4G). We further analyzed the genes of cell cycle involved in the G1/M and G2/S phases, and confirmed that they were relatively over-expressed with high proliferative activity at the embryonic period. Meanwhile, the cell cycle genes remained activity for a longer time in Landrace than in Tongcheng pigs at the embryonic period (S13 Fig). These results indicated that Landrace had a longer proliferation time and higher proliferation activity of myoblast than Tongcheng at the embryonic period. Meanwhile, the total number of myofibers (TNFs) is generally considered to be established and fixed at E90 in Western pigs [2,3]. We observed that the E80, E85 and E90 in Landrace clearly matched the same time points in Tongcheng pigs (Fig 4F). This fact suggested that there was similar myofiber development between the two breeds at these time points and the TNFs were mostly established before E90 in both Landrace and Tongcheng pigs. From birth to D140, muscle development in Landrace obviously lagged compared to Tongcheng pigs. This fact implied that muscle maturation occurs earlier in Tongcheng than in Landrace, consisting with the larger muscle fiber area of Landrace pigs.

### Genetic differentiation between Landrace and Tongcheng pigs

**Fig 5:**
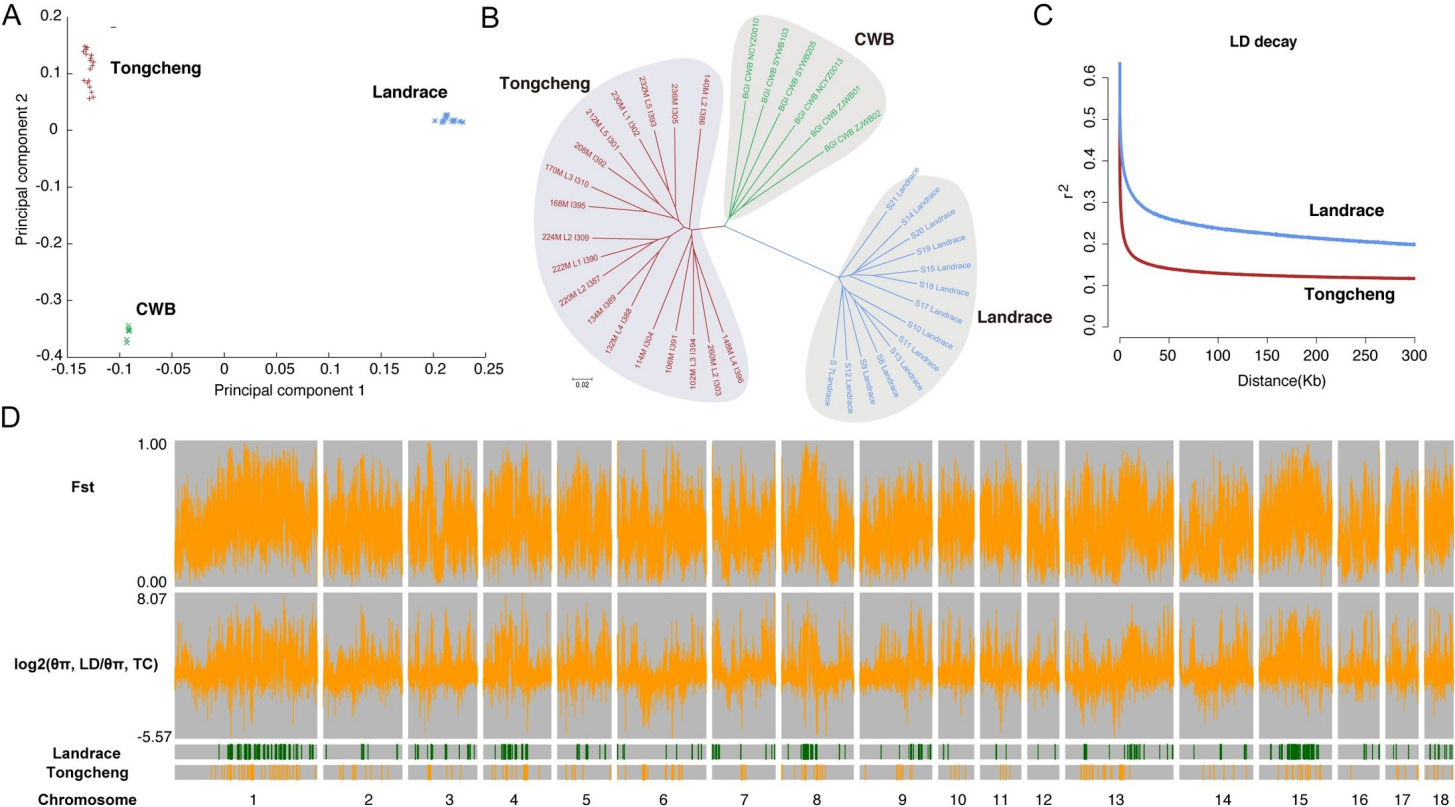
Genetic diversity between Landrace and Tongcheng pigs. (**A**) Principal components analysis (PCA) of 38 pigs from Tongcheng pigs, Landrace pig and Chinese wild boars. (**B**) A neighbor-joining phylogenetic tree constructed using SNP identified in the 38 pigs. (**C**) Linkage disequilibrium patterns of Landrace and Tongcheng pigs. (**D**) The distribution of the genome-wide *F*_*ST*_ (top), log_2_(θ_π, Landrace_/θ_π, Tongcheng_) values (middle) and selected regions (bottom) in Landrace and Tongcheng pigs across 18 autosomes.

The obese- and lean-type pigs have undergone different artificial and natural selection pressure during modern breeding, and resulted in great phenotypic differences [14]. To further understand the effect of genetic variations on phenotype differences in skeletal muscle, we re-sequenced the genome of 18 Tongcheng pigs with more than 10-fold depth per individual. Additionally, we downloaded the re-sequencing data of 14 Landrace pigs and six Chinese wild boars (CWB) from the NCBI Sequence Read Archive (SRA) database (S11 Table). A total of 26,641,976 single nucleotide polymorphisms (SNPs) and 3,742,123 insertion–deletion mutations (InDels) were identified. Based on SNPs, we firstly conducted the neighbor-joining phylogenetic and principal component analysis of Landrace, Tongcheng and CWB. Both analyses clearly separated the three breeds with each other (Fig 5A and 5B). Meanwhile, Tongcheng pigs were found to have faster linkage disequilibrium (LD) decay than Landrace pigs (Fig 5C). Population selection signals showed that 453 and 529 genes located in genomic regions with putative selective sweep signals in Tongcheng and Landrace pigs, respectively (Figs 5D and S14, and S12 Table). Enrichment analyses revealed that the selected genes in Tongcheng were significantly associated with biomineral formation, organic anion transport and ossification, while the selected genes in Landrace were significantly enriched in GO terms for cell motility, localization of cell, cell migration and phosphorylation, such as *EDNRB*, *VEGFC* and *CD34* (S13 Table). These results suggested that obviously genetic differentiation existed between Landrace and Tongcheng pigs, which potentially contributes to the phenotypic differences in skeletal muscle between the two breeds.

### Functional implication of selected genes in skeletal muscle development

**Fig 6:**
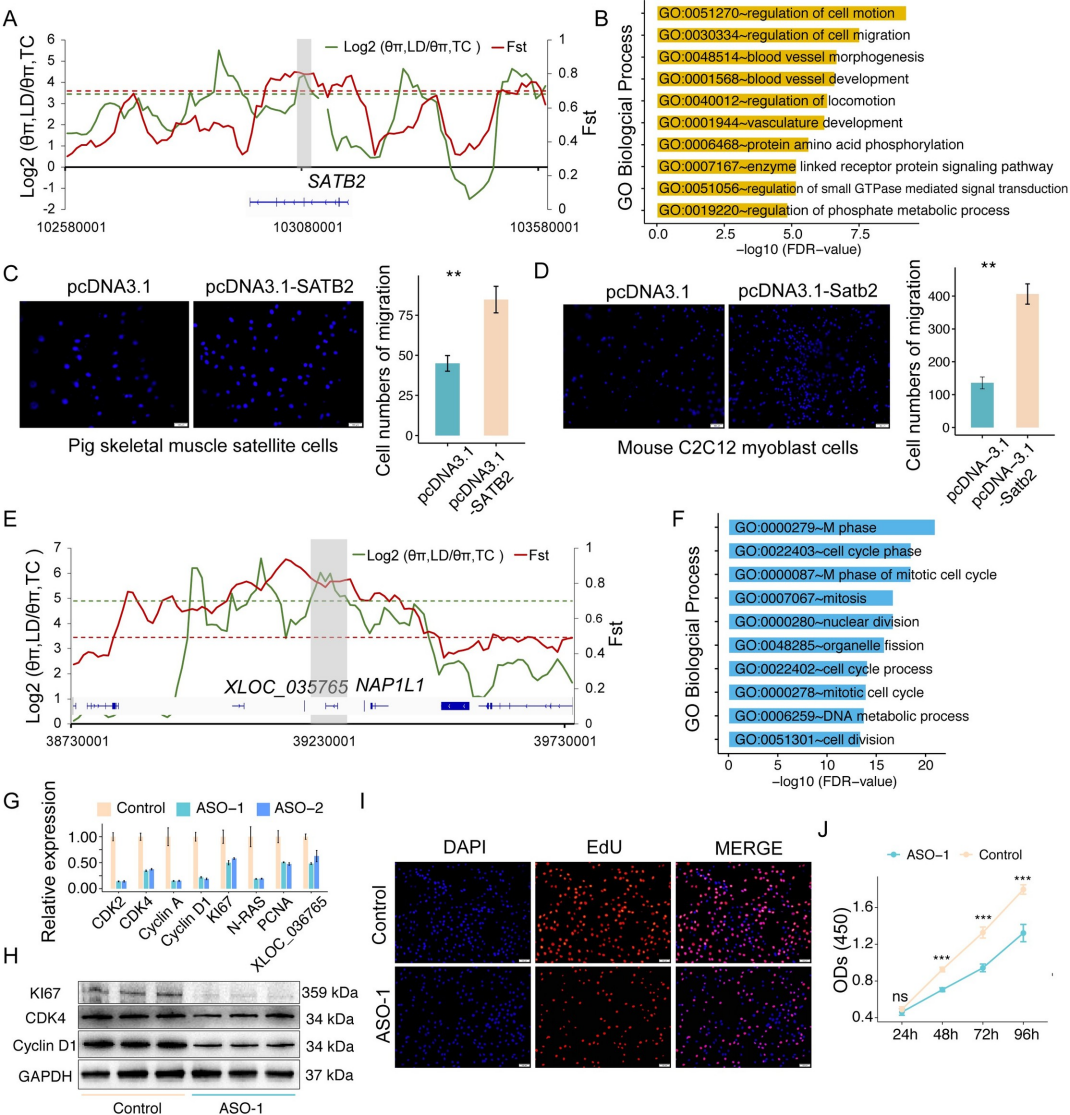
Function analysis of *SATB2* and *XLOC_036765*. (**A**) The θ ratio and *F*_*ST*_ values around the genomic region of the *SATB2* gene. (**B**) GO enrichment analysis of the genes co-expressed with *SATB2* during skeletal muscle development. (**C-D)** Transwell assay showing the change of migration ability after overexpressing *SATB2* in pig SkMCs (**C**) and mouse C2C12 myoblast cells (**D**). (**E**) The θ ratio and *F*_*ST*_ values around the *XLOC_036765*. (**F**) GO analysis of the genes co-expressed with *XLOC_036765* during skeletal muscle development. (**G-H**) qRT-PCR (**G**) and western blot (**H**) analysis of the expression levels of muscle proliferation and cell cycle markers following *XLOC_036765* knockdown in pig SkMCs. (**I**) EdU assay for proliferation of pig SkMCs following *XLOC_036765* knockdown. **(J)** CCK8 assay for the proliferation of pig SkMCs following *XLOC_036765* knockdown.

To further analyze the regulation role of genetic differentiation in the phenotypic diversity of skeletal muscle, we next explored whether the selected genes were differentially expressed (Type 1 and Type 2 DEGs) between Landrace and Tongcheng pigs during skeletal muscle development (S14 Table). The transcriptome analysis suggested that genes associated with cell migration and motility were up-regulated in Landrace pigs (S9 Table). We found that transcriptional factor *SATB2* had a selective sweep signal in Landrace (Fig 6A) and was up-regulated in Landrace at the adult stage. (S15A Fig). Interestingly, the *SATB2* was co-expressed with genes involved in cell migration (Pearson *R* > 0.5 and *P* < 0.001, Fig 6B), such as *TGFβ1*, *TGFBR3* and *SMAD7* [38]. To validate the role of *SATB2* in cell migration, we overexpressed *SATB2* in muscle cells. Transwell assay revealed that the *SATB2* overexpression obviously increased migration and invasion abilities of both pig SkMCs (Fig 6C) and mouse C2C12 myoblast cells (Fig 6D). Meanwhile, the functions of *SATB2* in myoblast proliferation and differentiation were also evaluated. The results showed that overexpression of *Satb2* promoted the proliferation and blocked the differentiation of C2C12 cells (S16 Fig). These results suggested the genetic selection of *SATB2* potentially contribute to skeletal muscle diversity between different breeds via affecting myoblast migration.

In addition to PCGs, we also found that 10 selected lincRNAs were differentially expressed between Landrace and Tongcheng pigs (S14 Table). Among these lincRNAs, *XLOC_036765* was specifically expressed at prenatal stage and had a selective-sweep region in Landrace pigs (Figs 6E and S15B). The *XLOC_036765* located at the upstream of nucleosome assembly protein 1 like 1 (*NAP1L1*), which plays a role in modulating chromatin formation and contributes to cell proliferation [39]. We found that 1,719 mRNAs were co-expressed with *XLOC_036765* (Pearson *R* > 0.5, *P* < 0.001). The functional enrichment analysis revealed that these mRNAs extensively involved in cell cycle (Fig 6F). Interestingly, the transcriptome analysis also suggested the genes associated with cell cycle were activated for a longer time in the embryonic skeletal muscles of Landrace (Fig 3G). To validate the effect of *XLOC_036765* in myoblast proliferation, we knocked it down by antisense oligonucleotides (ASOs) in porcine SkMCs. The result showed that *XLOC_036765* knockdown significantly decreased the expression of proliferation and cell cycle markers at both mRNA (Fig 6G) and protein (Fig 6H) levels. Meanwhile, both the ethynyl-2′-deoxyuridine (EdU) incorporation (Fig 6I) and CCK8 assay (Fig 6J) showed that *XLOC_036765* knockdown significantly decreased proliferation activity of myoblast cells. These results indicate *XLOC_036765* promotes myoblast proliferation in embryonic skeletal muscle through *cis* and/or *trans* mechanisms. Together, these findings suggested that the genetic differentiation in pigs affects the diversity of skeletal muscle development by regulating the expression of coding and non-coding RNAs.

## Discussion

Our systematic profiling of the dynamic transcriptome across 27 developmental time points and whole genome re-sequencing in Landrace and Tongcheng pigs highlight the role of genetic differentiation in the dynamic and divergent of skeletal muscle development. These results markedly improve our understanding of the dynamic transcriptional and genetic regulation of skeletal muscle development. This allows us to uncover genetics mechanisms of skeletal muscle diversity.

The dynamic transcriptome profile clearly indicates that the transcription complexity and molecular activity are highest prenatally and decrease as skeletal muscle development progresses, a result similar to that of the developmental processes of other tissues [40]. Widespread changes and period specificity of gene expression precisely capture the transcriptional characteristics of skeletal muscle development. For example, cell cycle and RNA splicing genes, expected during the busyness of the first waves of myogenesis proliferation, are specifically expressed in the embryonic period [15]. The activation of genes associated with muscle contraction and cell differentiation in the fetal period coincides with the second wave of myogenesis [4,20]. Neonatal transcriptional dynamics likely reflect the transition from a prenatal program of myogenesis to the differentiation of muscle fiber types and to metabolic maturation [41], since cellular respiration and oxidation-reduction processes are greatly activated at that period. Moreover, genes associated with glycogen metabolism are overrepresented in adult muscles, thus representing the skeletal muscle maturation processes [5]. Therefore, these results suggest that distinct transcriptional programs and biological processes are activated or depressed at specific stages, which provided valuable clues for understanding their functions in skeletal muscle development.

Compared to Tongcheng, Landrace pigs have been subject to decades of artificial selection aimed at creating faster growth and higher muscle mass [14,15]. Our data uncover a divergence in the developmental timing of gene expression and asynchronous development in skeletal muscle between those two breeds. The timing of certain events varies considerably between the two breeds during skeletal muscle development. Especially, the genes involved in the cell cycle are activated for a longer time in Landrace than in Tongcheng pigs in the embryonic period, indicating that Landrace pigs have longer time and higher activity of myoblast proliferation than Tongcheng pigs, and myoblasts are determined earlier in Tongcheng than in Landrace pigs during embryogenesis. This finding is in line with our previous observation of greater numbers of primary muscle fibers in Landrace pigs than in Tongcheng pigs [15]. Additionally, mitochondrial biogenesis gene expression in Tongcheng pigs is greater than in Landrace pigs in the postnatal stages. These differences indicate greater numbers of oxidative-type fibers in Tongcheng pigs, supporting the hypothesis that intensive selection for rapid growth in modern breeding has induced a shift in muscle metabolism toward a more glycolytic and less oxidative fiber type [42].

LncRNAs can function as competing endogenous RNAs (ceRNAs) that sponge miRNAs to regulate the miRNA target gene expression, thus regulating myogenesis and skeletal muscle development [7,43]. Based on our data, we identified two comprehensive ceRNA regulatory networks that showed opposing expression patterns potentially regulate skeletal muscle development. These findings expand our existing knowledge of coding and non-coding expression in pigs and provided a new strategy to elucidate the biological processes of skeletal muscle development. Another important finding from our study is that miR-24-3p involved insulin signaling pathway might drive the differences in glucose metabolism of adult skeletal muscle between Landrace and Tongcheng pigs. miR-24-3p is reported to be a negative regulator of mitochondrial function and insulin signaling pathway [35]. Two targets of miR-24-3p (*PRKAG3* and *PHKG*) have been well-known to play key roles in the regulation of glucose metabolism in skeletal muscle and affect pork meat quality [44,45]. The causal mutations in *PRKAG3* and *PHKG1* genes generate excess glycogen content in skeletal muscle [44,45]. These results highlight the critical role of miR-24-3p in regulating glucose metabolism and affecting meat quality, and the targets (mRNAs and lncRNAs) of miR-24-3p may be valuable candidates for further breeding in pork quality.

Natural selection and human-driven artificial selection have resulted in marked phenotypic diversity between Western commercial and Chinese local pig breeds [14,15]. The integrative analysis of transcriptome and genome re-sequencing can potentially shed light on genomic diversity and the genetic architecture in meat growth. It is noteworthy that Landrace pigs showed a higher expression of genes associated with cell cycle and cell migration than Tongcheng pigs. Interestingly, multiple selected genes in Landrace pigs, including TFs and lncRNAs, such as *SATB2* and *XLOC_036765*, are functionally related to cell migration and cell cycle. We reason that greater abilities of myoblast proliferation and cell migration in Landrace may be driven by the artificial selection during modern pig breeding, resulting in great differences in muscle growth between Western commercial and Chinese local pig breeds. However, further studies are needed to validate its regulation mechanism in regulating skeletal muscle development and growth.

## Conclusions

Overall, by integrating analysis of dynamic transcriptome over extended developmental periods and genome re-sequencing data, we elucidate the potential genetic basis underlying the transcriptome dynamic and diversity of skeletal muscle development in pigs, and report many candidate genes associated with muscle growth. These data and findings not only provide valuable resources for animal breeding, but also were helpful to understand muscle relevant diseases in humans.

## Materials and methods

### Ethics statement

All animal procedures followed protocols approved by the Hubei Province of China for Biological Studies Animal Care and Use Committee, the Chinese Academy of Agricultural Sciences and the Institutional Animal Care and Use Committee.

### Skeletal muscle collection

We collected *longissimus dorsi* muscle samples from Landrace and Tongcheng pigs at 27 developmental time points, including embryonic days (E33, E40, E45, E50, E55, E60, E65, E70, E75, E80, E85, E90, E95, E100, and E105) and postnatal days (D0, D9, D20, D30, D40, D60, D80, D100, D120, D140, D160, and D180). The animals were allowed access to food and water *ad libitum* and were housed under identical conditions. After copulation with a boar of each corresponding breed, the sows were sacrificed at a commercial slaughterhouse at the selected stages. At each time point, samples from three pigs were harvested as biological replicates and the body weights and lengths of piglets during each embryonic stage were recorded. All samples were frozen immediately in liquid nitrogen and stored there until further use. Same samples were used for RNA-seq and miRNA-seq analysis.

### RNA-seq library construction and sequencing

We extracted total RNA using Trizol reagent (Invitrogen, Carlsbad, CA, USA) according to the manufacturer’s instructions. At each developmental stage of each breed, equal amount of total RNA was pooled from three individual pigs for library construction. Genomic DNA was removed using DNase I enzyme. The quality and concentration of total RNA were determined by agarose gel electrophoresis and a 2100 Bioanalyzer (Agilent Technologies, Santa Clara, CA, USA) and acceptable total RNAs must have had RNA integrity numbers ≥ 7.0 and 28S:18S ratios ≥ 0.7. Those RNAs were used to construct RNA-seq libraries that were prepared following standard Illumina protocol. Briefly, polyadenylated RNA was isolated from total RNA using oligo–dT magnetic beads (Invitrogen, Carlsbad, CA, USA) and then fragmented into small pieces by divalent cations in a thermomixer at an elevated temperature. The RNA fragments were reverse-transcribed with random primers to synthesize first-strand cDNA. Then, second-strand cDNA was synthesized using DNA polymerase I with ribonuclease H (Invitrogen, Frederick, MD, USA). It then underwent end-repair, dA-tailing, and adapter ligation. Appropriately sized fragments were selected by agarose gel electrophoresis and then enriched by PCR amplification and examined by an Agilent 2100 Analyzer. Lastly, the prepared libraries were subjected to paired-end sequencing with 90-bp reads using an Illumina HiSeq 2000 sequencing system.

### Transcriptome data analysis

Reference genome sequences and gene annotation files were downloaded from Ensemble v95 and our RNA-seq reads were aligned to the pig reference genome (v11.1) [46] using TopHat v2.1.0 [47]. Gene annotation was used to guide read mapping and no more than 2 mismatches were allowed. RPKMs (reads per kilobase per million reads) were calculated to estimate gene expression levels using HTSeq (version 0.6.1) [48]. To visualize the relatedness of all samples, we combined expression profile correlations with MDS to reduce the complexity of the expression data and produce an intuitive visualization of global patterns. Based on their correlated expression patterns, we calculated the distances between each pair of all 54 transcriptome samples and performed classical multidimensional scaling to represent these distances in two dimensions. We used the ‘cmdscale’ to calculate these distances of the transposed log2-transformed RPKM matrix (after adding 1 to avoid undefined values) [49].

Two types of DEGs between Tongcheng and Landraces pigs were identified. Type 1 DEGs are genes differentially expressed between the two breeds at each of the four developmental phases, and were identified by DEseq2 v1.20.0 in R [50] using the cutoffs of | log2 FC | ≥ 1 and FDR ≤ 0.05. Type 2 DEGs are genes showing different expression trajectories between Landrace and Tongcheng pigs during development, and were identified using a multiple linear regression model of the development stages and breeds. Genes were considered significantly differentially expressed between breeds if they passed an FDR-corrected significance threshold of *P* < 0.05.

### LncRNA identification

Mapped reads identified by TopHat2 [47] were assembled into transcripts using Cufflinks v1.3.0 [51], with the assistance of known annotations, and merged into a consensus transcriptome using Cuffmerge [51]. The consensus transcriptome underwent multiple stages of filtering to identify lincRNAs. Single-exon, short transcripts (less than 200 nt) and those that overlapped known genes were filtered first. Two programs, Coding–Non-Coding Index (CNCI) [52] and Coding Potential Calculator (CPC) [53], were used to remove transcripts with coding potential. Transcripts with similarity to known proteins in the UniRef90 protein database (BLAST E-value less than 10^−5^) or whose corresponding translated protein sequences had a known protein-coding domain in the Pfam database v30.0 were also discarded. The remained transcripts were considered high confidence lincRNAs.

### miRNA-seq and data processing

Libraries of small RNAs were prepared using the TruSeq Small RNA Sample Prep Kit according to the manufacturer’s instructions (Illumina, San Diego, CA, USA). Then, purified cDNA fragments were directly sequenced using an Illumina HiSeq 2000 sequencing platform. After eliminating reads without a 3′ adapter or insert tags, reads with 5′ adapter contaminants or poly(A) tails, and fragments of less than 18 nt, high-quality clean reads were aligned to annotated pig miRNAs gathered from miRBase (release 23)[54] and the pig reference genome using miRDeep2 [55]. The expression level of each miRNA was calculated and normalized to transcripts per million (TPM).

### Co-expression network analysis

K-means method was used for co-expression analysis for skeletal muscle samples at 27 different time points in Landrace and Tongcheng pigs. Genes with coefficients of variation larger than 0.5 were retained for this analysis. The normalized expression values (*Z* scores) of genes at different time points were calculated using R ‘*scale*’ (with the ‘scale’ and ‘center’ options set to TRUE). The optimal cluster number was determined by the Figure of merit [56].

### ceRNA network construction

RNAhybrid v2.1.2 [57] and miRanda v3.3a [58] were used to predict the putative targets for both lncRNAs and mRNAs with default parameters (E value < -20). To select bona fide targets, we retained only the miRNA-target relationships supported by both tools. To stringently define lncRNA-associated competing triplets, we computed the Pearson correlation coefficient (*R*) of each RNA–RNA pair based on expression values, and required relations to simultaneously satisfy all the following criteria: negative correlation between miRNAs and lncRNAs (*R* < -0.5, *P* < 0.05), negative correlation between miRNAs and mRNAs (*R* < -0.5, *P* < 0.05), and positive correlation between lncRNAs and mRNAs (*R* > 0.5, *P* < 0.05). Finally, we identified 69,503 lncRNA-miRNA-mRNA interactions. The ceRNA networks were visualized by the Cytoscape package (https://cytoscape.org/). A ‘guilt-by-association’ strategy [59] was used to predict the potential functions of lncRNAs in the networks.

### Development-associated genes analysis

We detected DAGs for each time point by selecting genes with absolute RPKM values greater than 1.0 and relative expression level *Z*-scores greater than 1.5 (normalized RPKM across samples), which guaranteed that the selected DAGs were expressed at levels distinguishable from background noise and highly expressed in specific time points relative to other time points [37]. For each time point pair, we used hypergeometric testing to test the significance of the number of shared DAGs. The *P*-value (in which the null hypothesis is that the two samples have independent DAGs and are therefore unrelated) was adjusted by Bonferroni correction. The mapping score was defined as –log10 (Bonferroni-corrected *P*-value), implying similarity in their transcriptome characteristics. After recording the mapping scores of all pairwise comparisons in a matrix (M), we identified the maximum trace by maximizing the sum of the mapping scores of the time point pairs it passes through using dynamic programming. More specifically, to identify the maximum trace, for each time point pair (i, j), we computed the possible largest sum (from M (1, 1) to the current position M (i, j)). Then, we located M (27, 27) and traced back from the largest value to M (1, 1) to get the maximum trace. The time point pairs that the maximum trace passed through implied the possible mapping relationship of development between Landrace and Tongcheng pigs.

### Whole-genome sequencing and data processing

To identify genomic regions harboring artificial selection signatures in Landrace and Tongcheng pigs, we generated the genome sequencing of 18 Tongcheng pigs (no direct and collateral blood relationship within three generations). Genomic DNAs from the ear tissues of these pigs using the DNeasy Blood & Tissue Kit (Qiagen) according to the manufacturer’s instructions. Libraries were constructed according to the manufacture’s standard protocols (Illumina, San Diego, CA, USA). Sequencing was performed to generate 150-bp paired-end reads on the Illumina NovaSeq 6000 platform (Berry Genomics Co., Ltd., Tianjin, China). In addition, we downloaded the genome sequencing data of 14 Landrace pigs and six Chinese wild boars from NCBI SRA database.

The high quality paired-end reads were mapped to the *Sus Scrofa* reference genome (Ensembl 95) using BWA (v0.7.12) [60] with the parameter: ‘mem -t 4 -k 32 -M’. PCR or optical duplicates were removed using SAMtools (v1.3.1) [61]. We performed SNP calling using a UnifiedGenotyper approach as implemented in the package GATK (Genome Analysis Toolkit, v3.7-0-gcfedb67) [62]. To remove the potential false positive SNPs, SNPs with QD < 2.0 or FS > 60.0 or MQ < 20.0 or MQRankSum <–12.5 or ReadPosRankSum <–8.0 were filtered. Gene-based SNP annotation was performed according to the annotation of *Sus scrofa* reference genome (Ensembl 95) using the package ANNOVAR (v2013-06-21) [63].

### Phylogenetic, population genetic and LD analyses

To analyze the population structure, we screened a subset of bi-allelic and high-quality SNPs with a call rate ≥ 90% and a minor allele frequency ≥ 5%. A neighbor-joining tree was constructed using the program TreeBeST (v1.92, http://treesoft.sourceforge.net/treebest.shtml) with 200 bootstrap replicates and was displayed using MEGA5 [64]. To infer the population structure, we used ADMIXTURE (v1.3.0) [65], which implements a block-relaxation algorithm. To make consideration for HWE violations, we also filtered SNPs by testing HWE violations (*P* > 10^−4^) and reconstructed the model-based clustering analysis. Principal component analysis (PCA) was performed using the program GTAC (v1.92) [66]. To estimate and compare the pattern of linkage disequilibrium (LD) of Landrace and Tongcheng pigs, the squared correlation coefficient (*r*^*2*^) values between any two SNPs within 300 kb were computed using the software Haploview (v4.269) [67]. We produced an LD decay plot that shows the average *r*^*2*^ values in a bin of 100 bp against the physical distance of pairwise bins.

### Selective sweep analysis

To identify potential regions and genes under selection in Landrace and Tongcheng pigs, we removed SNPs with minor allele frequency below 0.05. θ_π,Landrace_/θ_π,Tongcheng_ and *F*_*ST*_ were calculated using VCFtools (v0.1.13) [68] with a 50 kb sliding window and a step size of 10 kb. Windows that contained less than 10 SNPs were excluded from further analysis. The windows that were simultaneously (1) in the top 5% of *F*_*ST*_ values (> 0.7) and (2) in the top and bottom 5% log2(θ_π, Landrace_/θ_π, Tongcheng_) (>3.43 or < -0.69) were considered to be candidate selective regions. The genes in the merged candidate selective regions along the pig genome were considered as selected genes.

### Function enrichment analysis

We performed GO and KEGG pathway enrichment using the Database for Annotation, Visualization, and Integrated Discovery (DAVID) v6.8 (http://david.abcc.ncifcrf.gov/) [69]. For the transcriptome analysis, including co-expression and differential expression analyses, all the expressed PCGs in skeletal muscle (n = 14,743) were used as as the background reference set. For the genome analysis, we used all the PCGs in the pig genome (n = 22,342) as the background reference set. *P*-values were adjusted to control the false discovery rate (FDR) using the Benjamini-Hochberg method.

### RNA interference and plasmid construction

Two ASOs specifically targeting *XLOC_036765* were synthetized by RiboBio (Guangzhou, China). The ASO with the higher interfering efficacy was used for the next study. To generate *SATB2* overexpression vector, the coding sequence regions of *SATB2* gene in pig and mouse were amplified using forward and reverse primers containing *BamH I* and *Xho I* sites, respectively. The PCR products were inserted into the pcDNA3.1(+) vector (Invitrogen). ASOs and overexpression vectors were transfected into pig SkMCs or mouse C2C12 cells to detect the effect of genes on myogenesis. The ASO and primer sequences are in S15 Table.

### Cell culture, proliferation, and differentiation

Pig SkMCs s and mouse myoblast C2C12 cells were maintained in our laboratory. The cells were cultured in DMEM (Sigma-Aldrich, St. Louis, MO, USA) supplemented with 10% fetal bovine serum (FBS) and 1% penicillin/streptomycin at 37°C in 5% CO_2_. We examined cell proliferation using the Cell Counting Kit-8 (CCK-8) (Dojindo, Kumamoto, Japan) and Cell-Light EdU DNA Cell Proliferation Kit (RiboBio, Guangzhou, China), as described previously [8]. For myogenic differentiation, the culture medium was switched to DMEM containing 2% horse serum for 2 days and then maintained in the culture medium for another 4 days.

### Luciferase reporter assay

To validate the targets of miR-24-3p, chemically synthesized miR-24-3p or the negative control duplexes (Gene Pharma, Shanghai, China) were transfected into the pig SkMCs in combination with a luciferase reporter containing wild-type or mutated 3′-UTR of target genes. All the co-transfection assays were performed in 12-well plates with Lipofectamine 3000 reagent (Invitrogen) according to the manufacturer’s instructions. After 48 h of incubation, the activities of Renilla and firefly luciferase were measured with the Dual Luciferase Assay System (Promega).

### Quantitative real-time PCR (qRT-PCR)

Total RNA was isolated using TRIzol reagent (Invitrogen), and cDNA was synthesized with a RevertAid First Strand cDNA synthesis kit (Thermo Fisher Scientific). The qPCR reaction was performed in triplicate using the SYBR Green Master Mix instructions (Applied Biosystems) on a 7500 FAST Real-Time PCR System (Applied Biosystems) according to the manufacturer’s instructions. Gene expression levels were normalized to the housekeeping gene *GAPDH* using the 2^-△△Ct^ method. The primer sequences are in S15 Table.

### Transwell assay

To validate the function of *SATB2* on cell migration, *SATB2* overexpression vector were transfected in pig SkMCs and mouse C2C12 cells. The cells were digested at 48 h after transfection and seeded into the upper chamber of a 24-well transwell (Corning, USA) which was precoated with 100μL serum-free DMEM/F12 medium for 10 minutes. DMEM/F12 medium with 20% FBS (500 μL) was then added to the lower chamber of transwell. After 12 h of incubation at 37°C in 5% CO_2_, the cells on the upper surface of the filter were removed with a cotton swab, the migrated cells on the lower surface of the transwell upper chambers were fixed with 4% formaldehyde solution for 15 min and stained with 10ug/mL DAPI for 10 min. The migrated cells were counts by counting 3 random high-power fields per filter under an inverted microscope (Olympus).

## Supporting information

S1 Fig Phenotype information of Landrace and Tongcheng pigs. Body weight (**A**) and length (**B**) of embryo or fetus from Landrace and Tongcheng pigs at E33, E65, E90 and E105 stages. (**C**) The ultrastructure of skeletal muscle of Landrace (*left*) and Tongcheng (*right*) pigs at E65 stage under 20,000 × electronic microscope. A, B and C in the pictures represent chondriosome, glycogenosome and nucleus, respectively. (**D**) Performance test of Landrace and Tongcheng pigs. Abbreviation: DT, days during test; LW, live weight; LBF, live backfat thickness; ABF, average backfat thickness at 3 points; 6-7^th^ BF, backfat thickness (depth) between 6^th^ and 7^th^ ribs; 10^th^ BF, 10^th^ rib backfat thickness; EMA, eye-muscle area; ADG1, average daily gain from birth to market; ADG2, average gain during the trial; IMF, intramuscular fat content; FCR, feed conversion ratio.
(TIF)

S2 Fig The molecular characteristics of identified lncRNAs. (**A**) Transcript length of lncRNAs and mRNAs. (**B**) Exon number of lncRNAs and mRNAs. (**C**) The coefficient of variation of the expression level of lncRNAs and mRNAs. (**D**) The expression level of lncRNAs and mRNAs.
(TIF)

S3 Fig Analysis of global gene expression during skeletal muscle development in Landrace and Tongcheng pigs. (**A**-**C**) Number of expressed mRNA (**A**), lncRNA (**B**) and miRNAs (**C**) at 27 developmental time points. (**D**) The gene expression abundances during skeletal muscle development. The y axis is z-scaled RPKM values; Dark lines represent median expression levels, and confidence bands represent 25th–75th percentiles of expression level for Tongcheng and Landrace pigs. (**E**) Expression patterns of the *MKI67* and *MYF4* genes during skeletal muscle development. (**F**) Coefficient of variation (CV) distribution for the expression levels of detected genes.
(TIF)

S4 Fig Differentially expressed genes (DEGs) between adjacent consecutive periods during skeletal muscle development. (**A-C**) Volcano plot showing the DEGs between fetal and embryonic periods (**A**), between neonatal and fetal periods (**B**), and between adult and neonatal periods (**C**). NS, not significant. (**D-F**) Top enriched GO terms of the down- and up-regulated genes between fetal and embryonic periods (**D**), between neonatal and fetal periods (**E**), and between adult and neonatal periods (**F**).
(TIF)

S5 Fig Analysis of the expression correlation (Pearson) between *MYOG* and *YBX3* during skeletal muscle development. (TIF)

S6 Fig The expression of IGFs and their receptors and binding proteins during skeletal muscle development in Landrace and Tongcheng pigs. (TIF)

S7 Fig Co-expression analysis. (**A**) Module 5 (M5). Left, the median Z score of genes in M8 across skeletal muscle development, Right, the top 10 typical GO biological process terms associated with M5. (**B, C**) Same analyses as in A, but for M8 and M9. Note: no GO term reached the significance threshold (FDR < 0.05) in M8.
(TIF)

S8 Fig ceRNA network. (**A**) An integrative pipeline for transcriptome-wide identification of lincRNA-miRNA-mRNA regulatory networks. Interactions between miRNAs and their targets are predicted using three computational approaches (miRanda, PITA and RNAhybrid). miRNA-lincRNA and miRNA-mRNA pairs sharing the same miRNAs are merged into a lincRNA-miRNA-mRNA interaction network as a candidate ceRNA network. Pig miRNAs and mRNA annotations are obtained from miRBase and the Ensembl database, respectively. LincRNAs are identified using our transcriptome dataset with multiple filter steps. (**B**) Heatmap showing the expression of lincRNAs and mRNAs in network I and II during skeletal muscle development.
(TIF)

S9 Fig The expression of miR-24-3p during skeletal muscle development in Landrace and Tongcheng pigs. (TIF)

S10 Fig Interactive graph showing GO enrichment analysis of genes showing different expression trajectories between Landrace and Tongcheng pigs. (TIF)

S11 Fig Heatmap showing the relative expression patterns of the NADH dehydrogenase and mitochondrial genes during skeletal muscle development in Landrace and Tongcheng pigs. (TIF)

S12 Fig Number of developmental-associated genes in Landrace and Tongcheng pigs at each time point. (TIF)

S13 Fig Heatmap showing the relative expression patterns of the cell cycle genes during skeletal muscle development in Landrace and Tongcheng pigs. (TIF)

S14 Fig Distribution of log2(θ_π_, Landrace/θ_π_, Tongcheng) ratios and *F*_*ST*_ values, which are calculated in 50-kb windows sliding in 10-kb steps. Data points located to the left and right of the left and right vertical dashed lines, respectively (corresponding to the 5% left and right tails of the empirical ratio distribution, where the ratios are -0.69 and 3.43, respectively), and above the horizontal dashed line (the 5% right tail of the empirical *F*_*ST*_ distribution, where *F*_*ST*_ is 0.7) were identified as selected regions for Landrace pigs (red points) and Tongcheng pigs (blue points), respectively.
(TIF)

S15 Fig The expression of *SATB2* (A) and *XLOC_036765* (B) at the four developmental phases in Landrace and Tongcheng pigs. **FDR ≤ 0.01 and log2|FC| ≥ 1, ***FDR ≤ 0.001 and |log2 FC| ≥ 1.
(TIF)

S16 Fig The function of *Satb2* in myoblast proliferation and differentiation. (***A***) EdU assay for proliferation of C2C12 myoblast cells following *Satb2* overexpression. (**B-C**) qRT-PCR (**B**) and western blot (**C**) analysis of the expression levels of muscle proliferation markers following *Satb2* overexpression in C2C12 cells. ***(*D*)*** Immunofluorescence assay for the differentiation of C2C12 myoblast cells following *Satb2* overexpression. (**E-F**) qRT-PCR (**E**) and western blot (**F**) analysis of the expression levels of muscle differentiation markers following *Satb2* overexpression in C2C12 cells.
(TIF)

S1 Table Summary of read mapping in the RNA-seq libraries. (XLSX)

S2 Table Summary of read mapping in the miRNA-seq libraries. (XLSX)

S3 Table Annotation of the identified lncRNAs. (XLSX)

S4 Table DEGs between adjacent phases. (XLSX)

S5 Table Phase-specifically expressed genes during skeletal muscle development. (XLSX)

S6 Table Genes in each co-expression module. (XLSX)

S7 Table LncRNA-miRNA-mRNA interactions in the ceRNA networks. (XLSX)

S8 Table Type 1 DEGs. (XLSX)

S9 Table GO enrichment analysis of the Type 1 DEGs between Landrace and Tongcheng pigs at the adult period. (XLSX)

S10 Table Type 2 DEGs. (XLSX)

S11 Table Sample information of whole-genome sequencing data used in the study. (XLSX)

S12 Table Genes that are in genomic regions with putative selective sweep signals in Tongcheng and Landrace pigs. (XLSX)

S13 Table GO enrichment analysis of the selected genes in Landrace and Tongcheng pigs. (XLSX)

S14 Table DEGs between Landrace and Tongcheng pigs that are in genomic regions with strong selective sweep signals. (XLSX)

S15 Table Primer information used in the study. (XLSX)
