# Supplementary figures and images for: The genome variation and developmental transcriptome maps reveal genetic differentiation of skeletal muscle in pigs
Source: PLoS Genet. 2021 Nov 15;17(11):e1009910. doi: 10.1371/journal.pgen.1009910 (PMC8629385; doi:10.1371/journal.pgen.1009910)

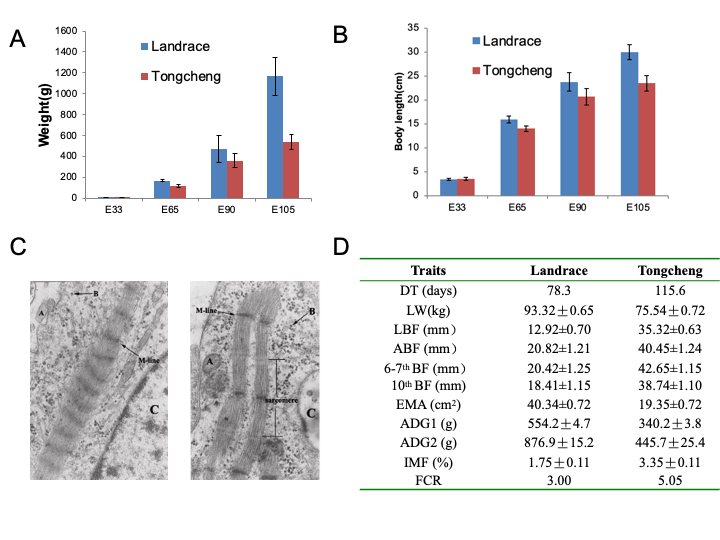

Supplement: S1 Fig — Body weight (A) and length (B) of embryo or fetus from Landrace and Tongcheng pigs at E33, E65, E90 and E105 stages. (C) The ultrastructure of skeletal muscle of Landrace (left) and Tongcheng (right) pigs at E65 stage under 20,000 × electronic microscope. A, B and C in the pictures represent chondriosome, glycogenosome and nucleus, respectively. (D) Performance test of Landrace and Tongcheng pigs. Abbreviation: DT, days during test; LW, live weight; LBF, live backfat thickness; ABF, average backfat thickness at 3 points; 6-7th BF, backfat thickness (depth) between 6th and 7th ribs; 10th BF, 10th rib backfat thickness; EMA, eye-muscle area; ADG1, average daily gain from birth to market; ADG2, average gain during the trial; IMF, intramuscular fat content; FCR, feed conversion ratio. (TIF) [file pgen.1009910.s001.tif]

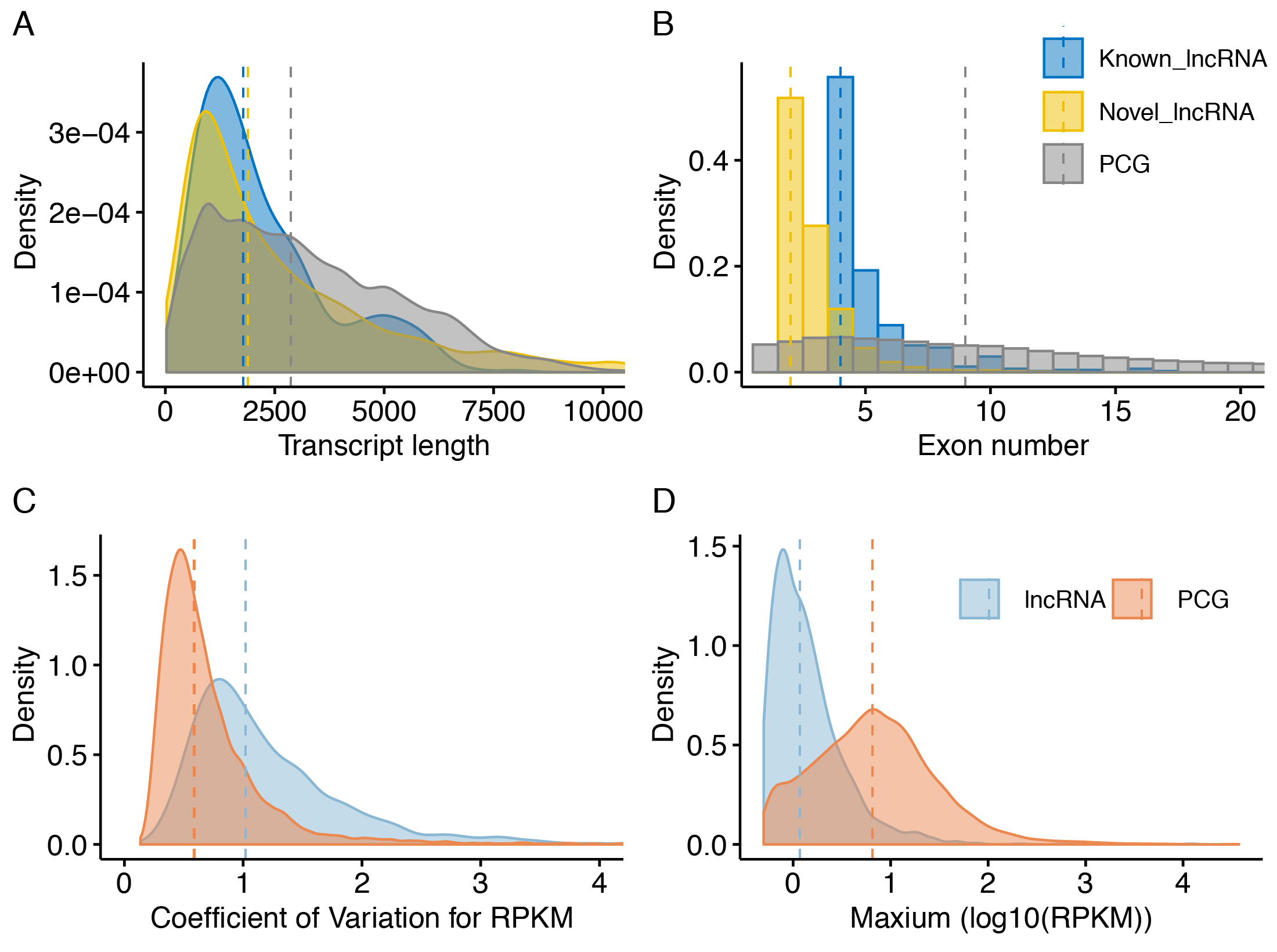

Supplement: S2 Fig — (A) Transcript length of lncRNAs and mRNAs. (B) Exon number of lncRNAs and mRNAs. (C) The coefficient of variation of the expression level of lncRNAs and mRNAs. (D) The expression level of lncRNAs and mRNAs. (TIF) [file pgen.1009910.s002.tif]

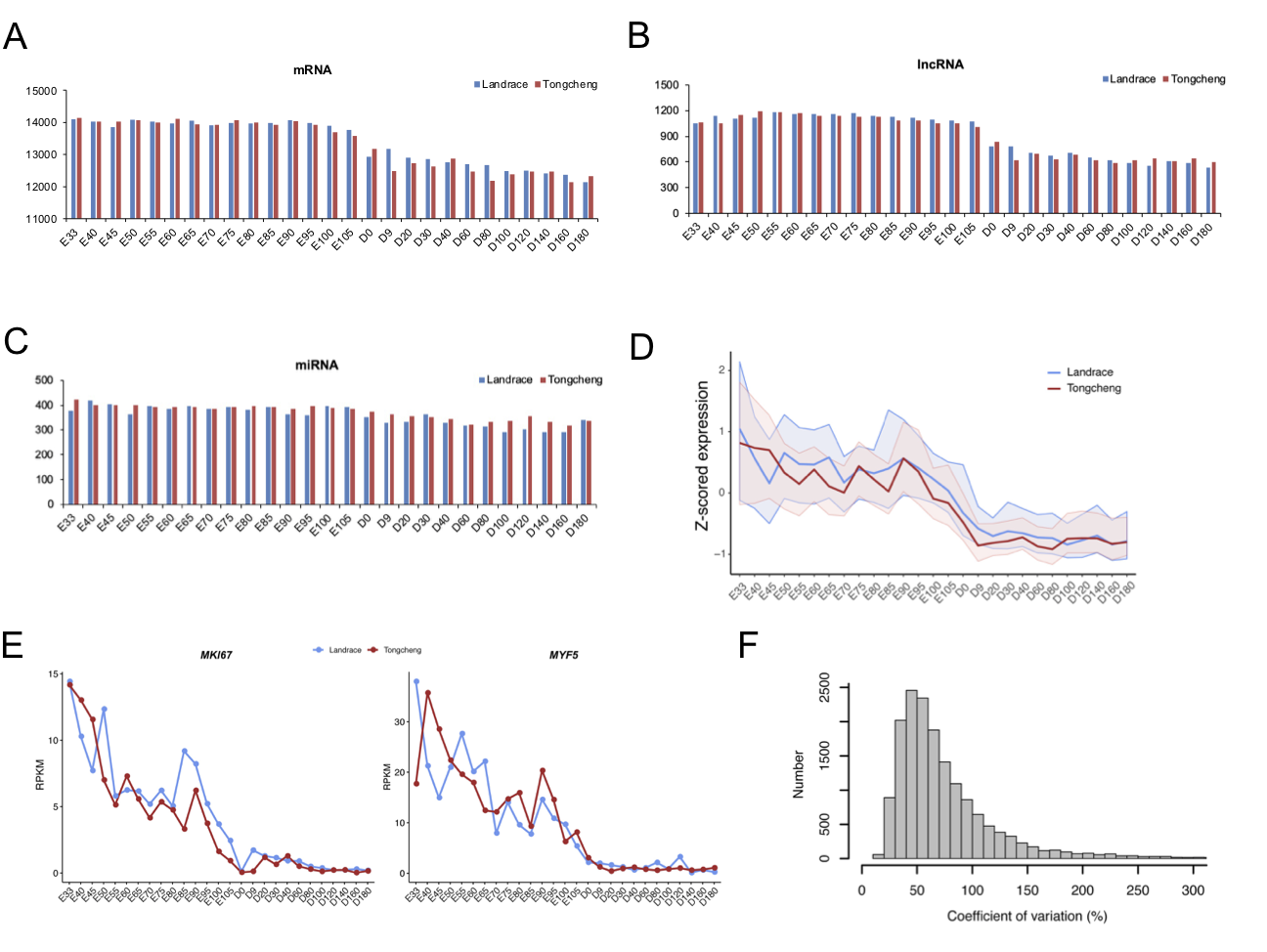

Supplement: S3 Fig — (A-C) Number of expressed mRNA (A), lncRNA (B) and miRNAs (C) at 27 developmental time points. (D) The gene expression abundances during skeletal muscle development. The y axis is z-scaled RPKM values; Dark lines represent median expression levels, and confidence bands represent 25th–75th percentiles of expression level for Tongcheng and Landrace pigs. (E) Expression patterns of the MKI67 and MYF4 genes during skeletal muscle development. (F) Coefficient of variation (CV) distribution for the expression levels of detected genes. (TIF) [file pgen.1009910.s003.tif]

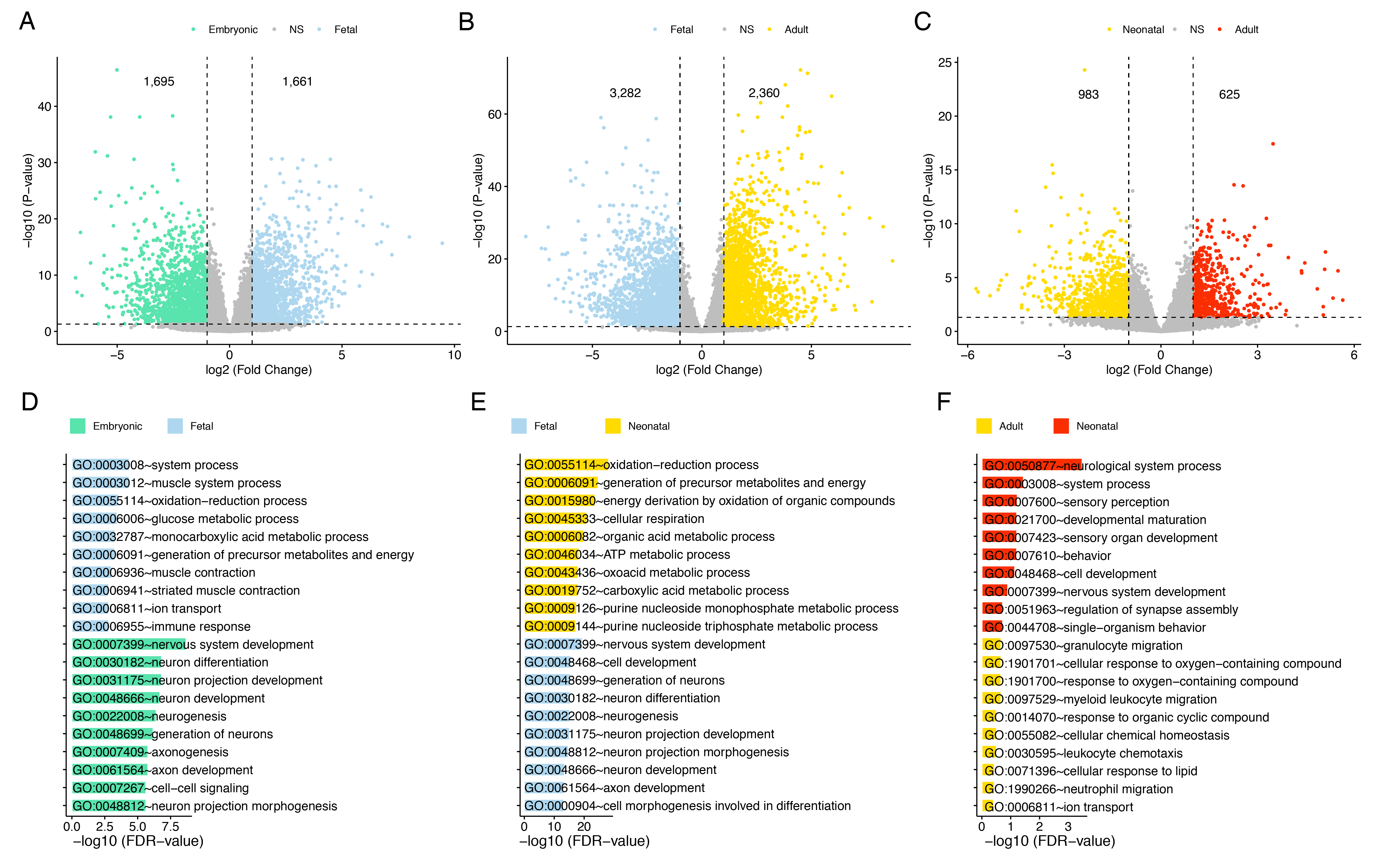

Supplement: S4 Fig — (A-C) Volcano plot showing the DEGs between fetal and embryonic periods (A), between neonatal and fetal periods (B), and between adult and neonatal periods (C). NS, not significant. (D-F) Top enriched GO terms of the down- and up-regulated genes between fetal and embryonic periods (D), between neonatal and fetal periods (E), and between adult and neonatal periods (F). (TIF) [file pgen.1009910.s004.tif]

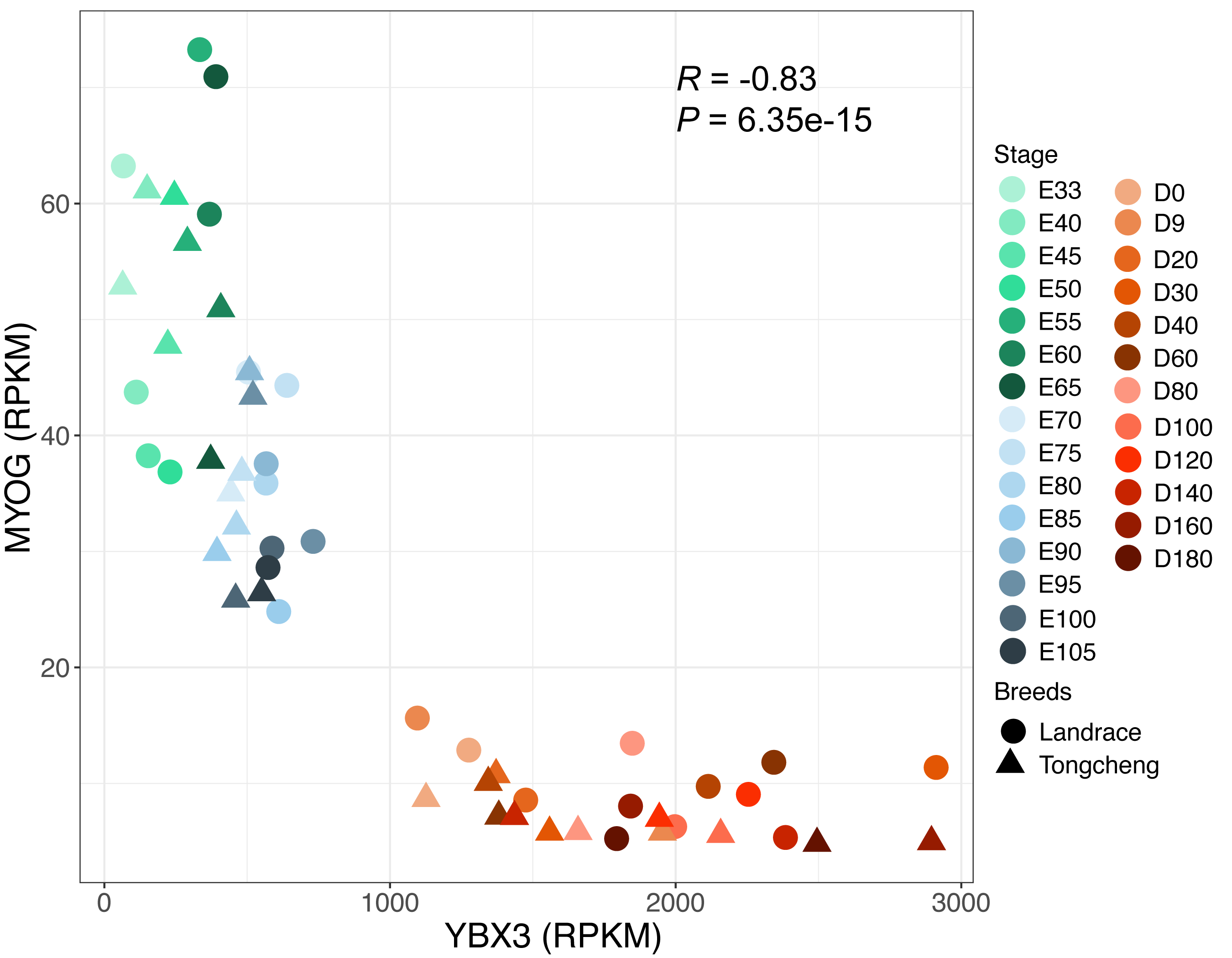

Supplement: S5 Fig — (TIF) [file pgen.1009910.s005.tif]

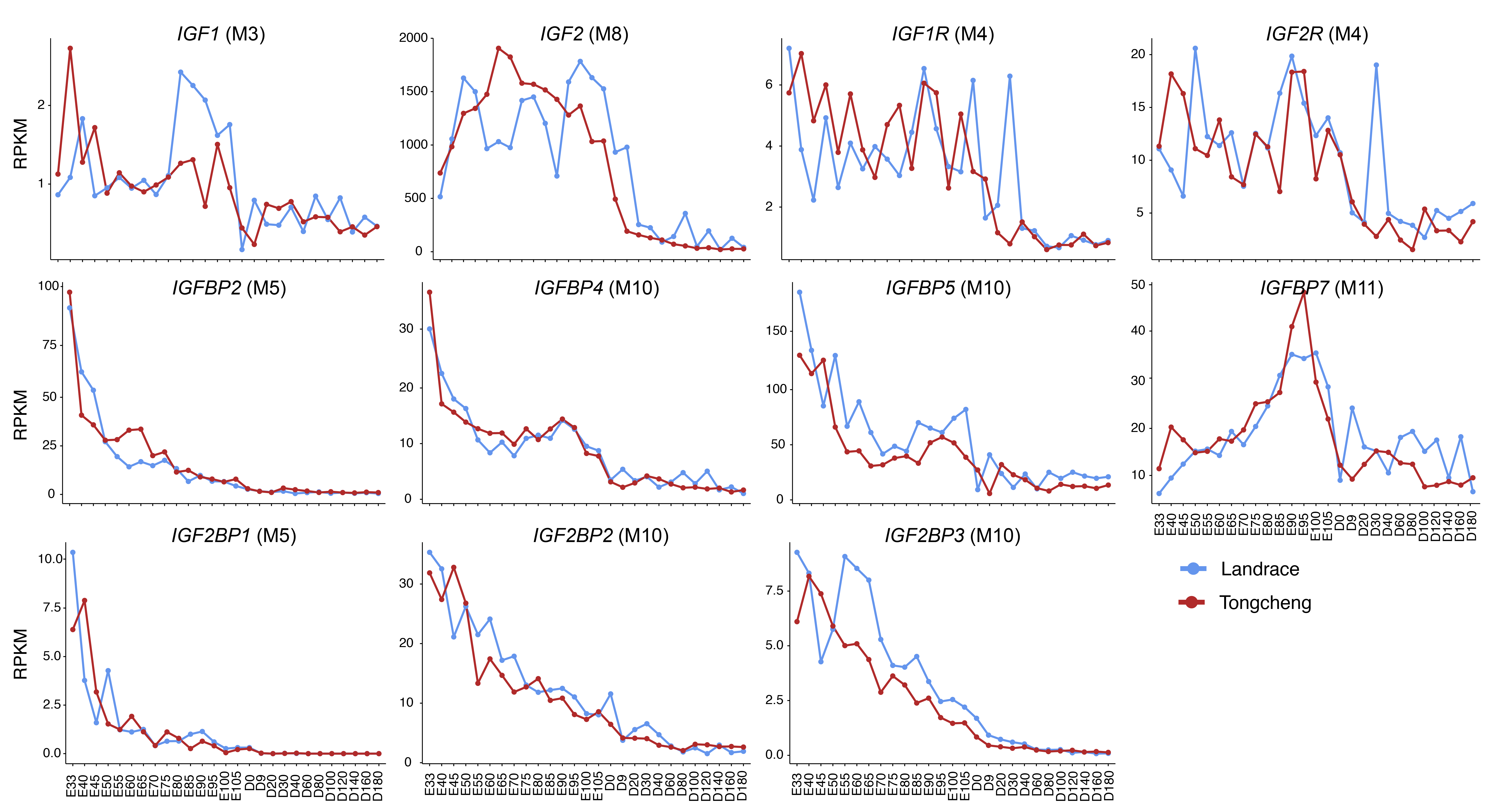

Supplement: S6 Fig — (TIF) [file pgen.1009910.s006.tif]

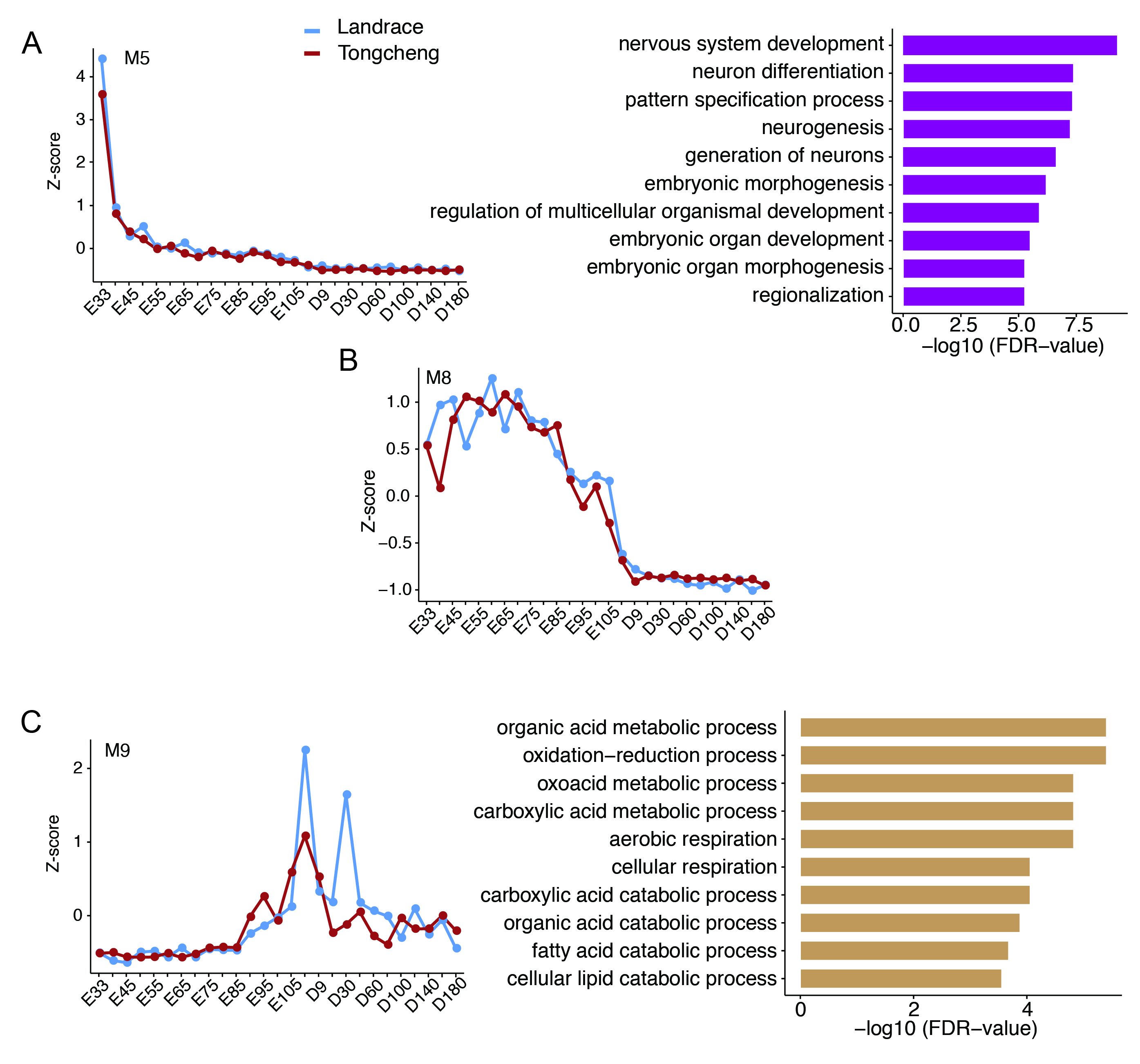

Supplement: S7 Fig — (A) Module 5 (M5). Left, the median Z score of genes in M8 across skeletal muscle development, Right, the top 10 typical GO biological process terms associated with M5. (B, C) Same analyses as in A, but for M8 and M9. Note: no GO term reached the significance threshold (FDR < 0.05) in M8. (TIF) [file pgen.1009910.s007.tif]

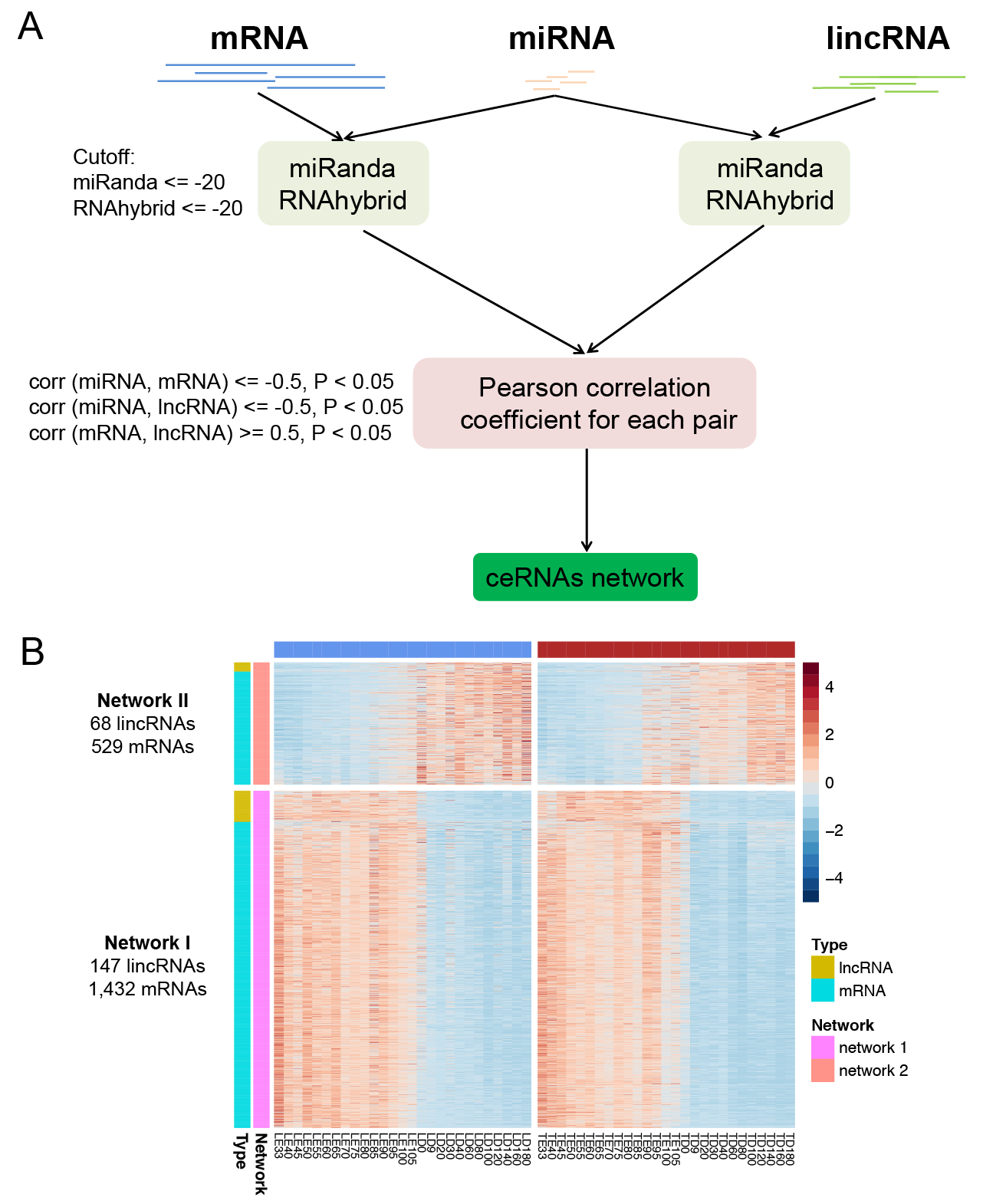

Supplement: S8 Fig — (A) An integrative pipeline for transcriptome-wide identification of lincRNA-miRNA-mRNA regulatory networks. Interactions between miRNAs and their targets are predicted using three computational approaches (miRanda, PITA and RNAhybrid). miRNA-lincRNA and miRNA-mRNA pairs sharing the same miRNAs are merged into a lincRNA-miRNA-mRNA interaction network as a candidate ceRNA network. Pig miRNAs and mRNA annotations are obtained from miRBase and the Ensembl database, respectively. LincRNAs are identified using our transcriptome dataset with multiple filter steps. (B) Heatmap showing the expression of lincRNAs and mRNAs in network I and II during skeletal muscle development. (TIF) [file pgen.1009910.s008.tif]

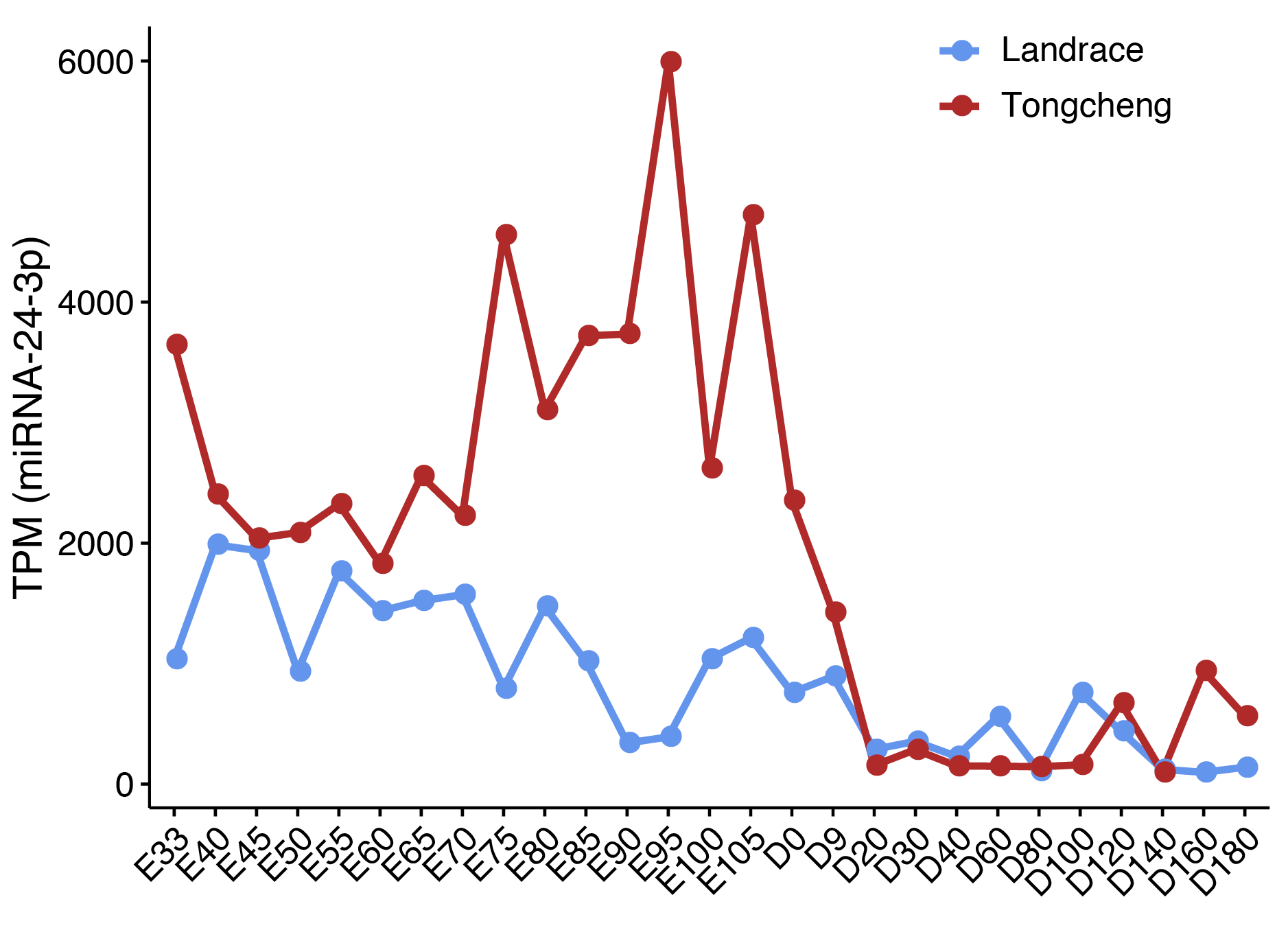

Supplement: S9 Fig — (TIF) [file pgen.1009910.s009.tif]

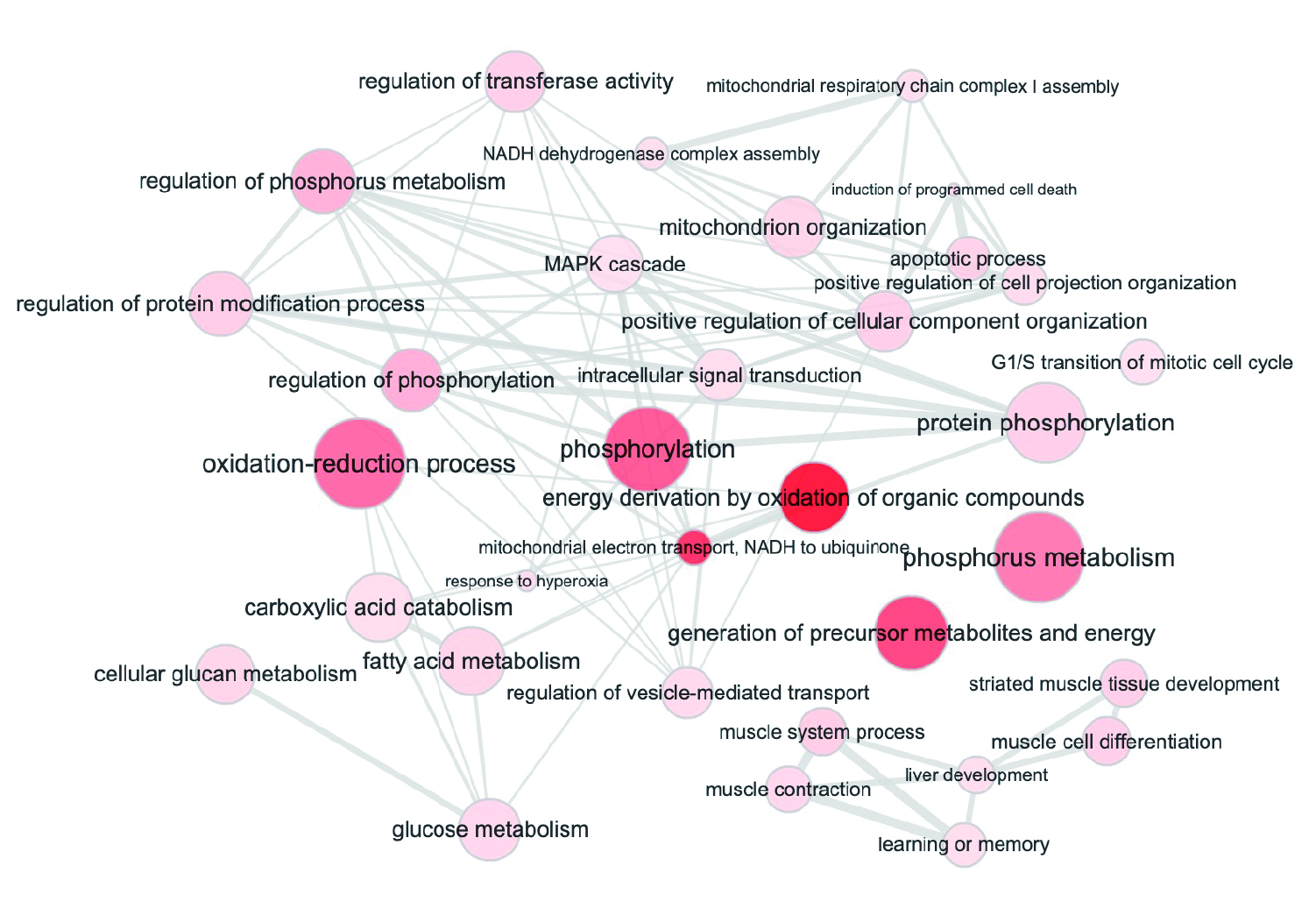

Supplement: S10 Fig — (TIF) [file pgen.1009910.s010.tif]

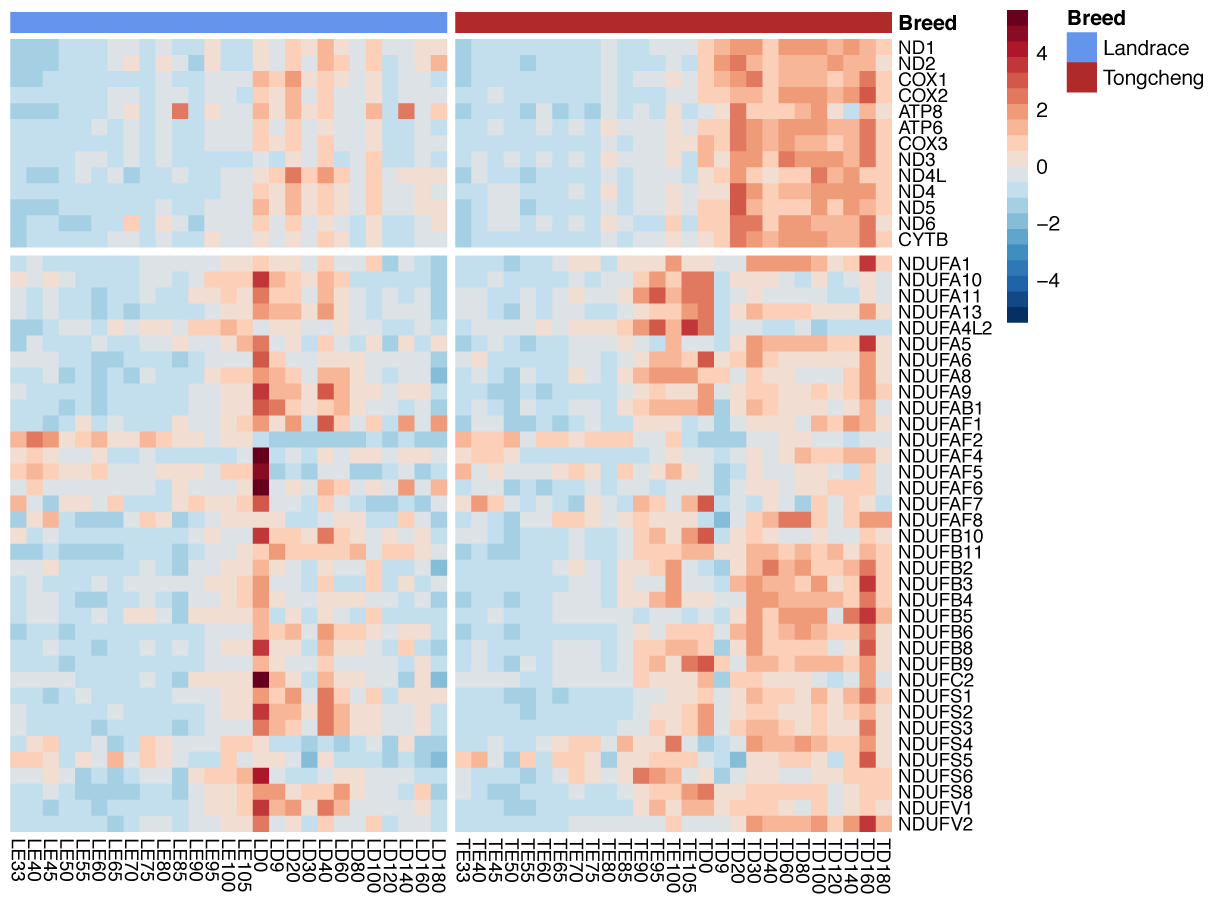

Supplement: S11 Fig — (TIF) [file pgen.1009910.s011.tif]

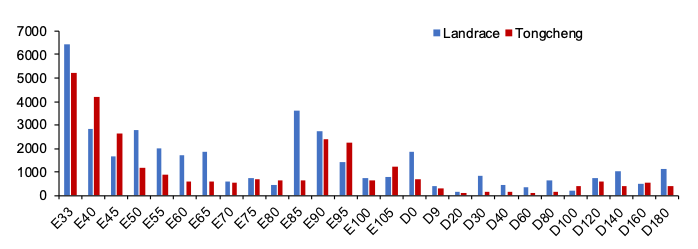

Supplement: S12 Fig — (TIF) [file pgen.1009910.s012.tif]

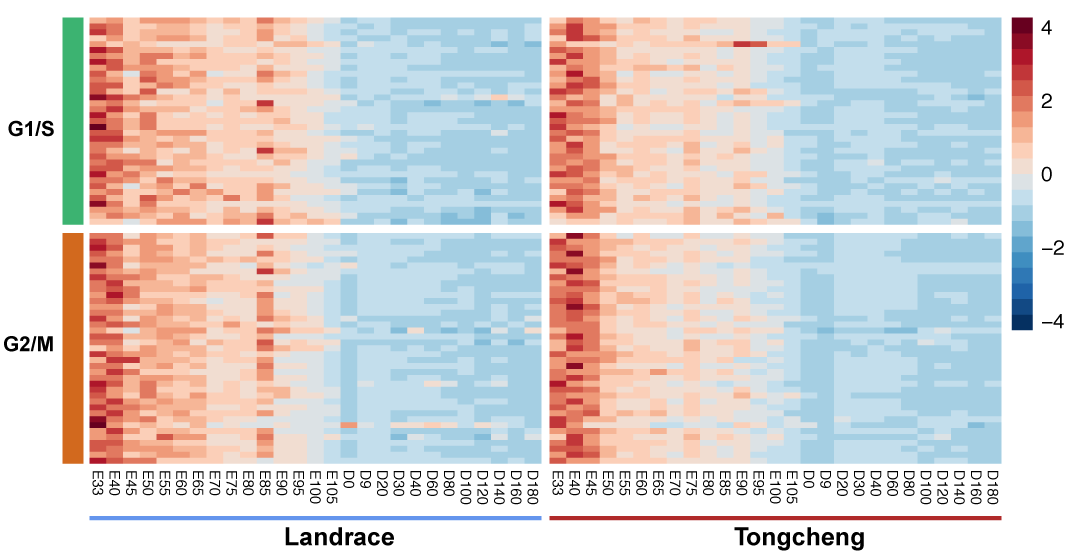

Supplement: S13 Fig — (TIF) [file pgen.1009910.s013.tif]

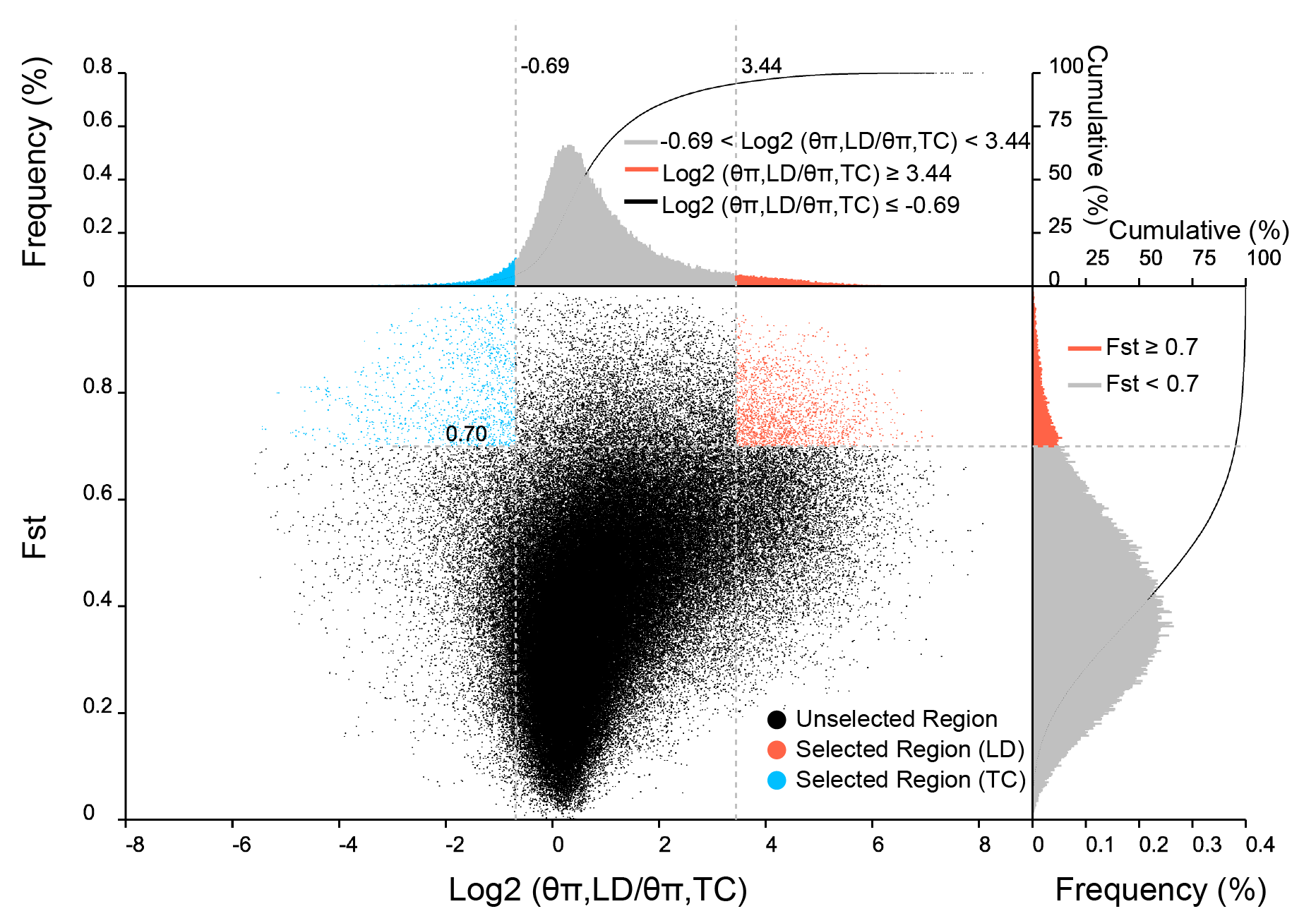

Supplement: S14 Fig — Data points located to the left and right of the left and right vertical dashed lines, respectively (corresponding to the 5% left and right tails of the empirical ratio distribution, where the ratios are -0.69 and 3.43, respectively), and above the horizontal dashed line (the 5% right tail of the empirical FST distribution, where FST is 0.7) were identified as selected regions for Landrace pigs (red points) and Tongcheng pigs (blue points), respectively. (TIF) [file pgen.1009910.s014.tif]

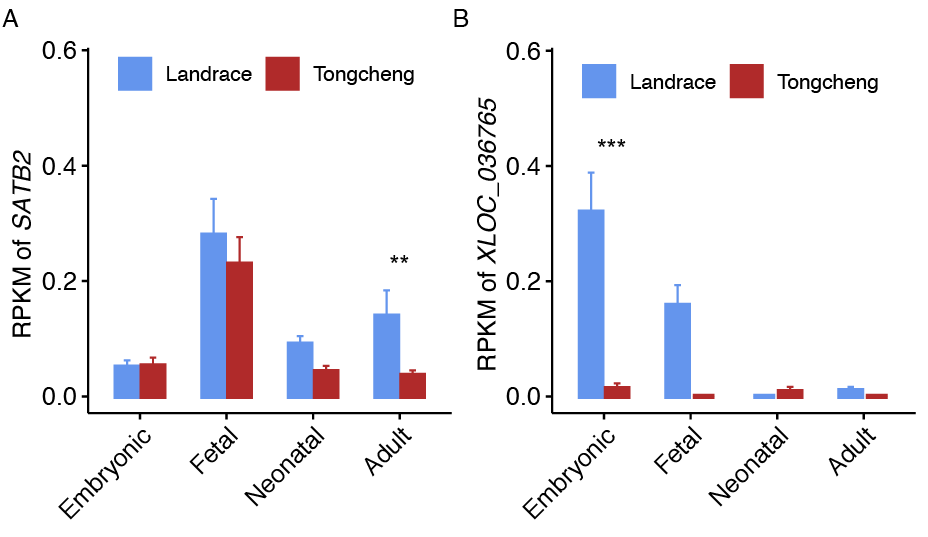

Supplement: S15 Fig — **FDR ≤ 0.01 and log2|FC| ≥ 1, ***FDR ≤ 0.001 and |log2 FC| ≥ 1. (TIF) [file pgen.1009910.s015.tif]

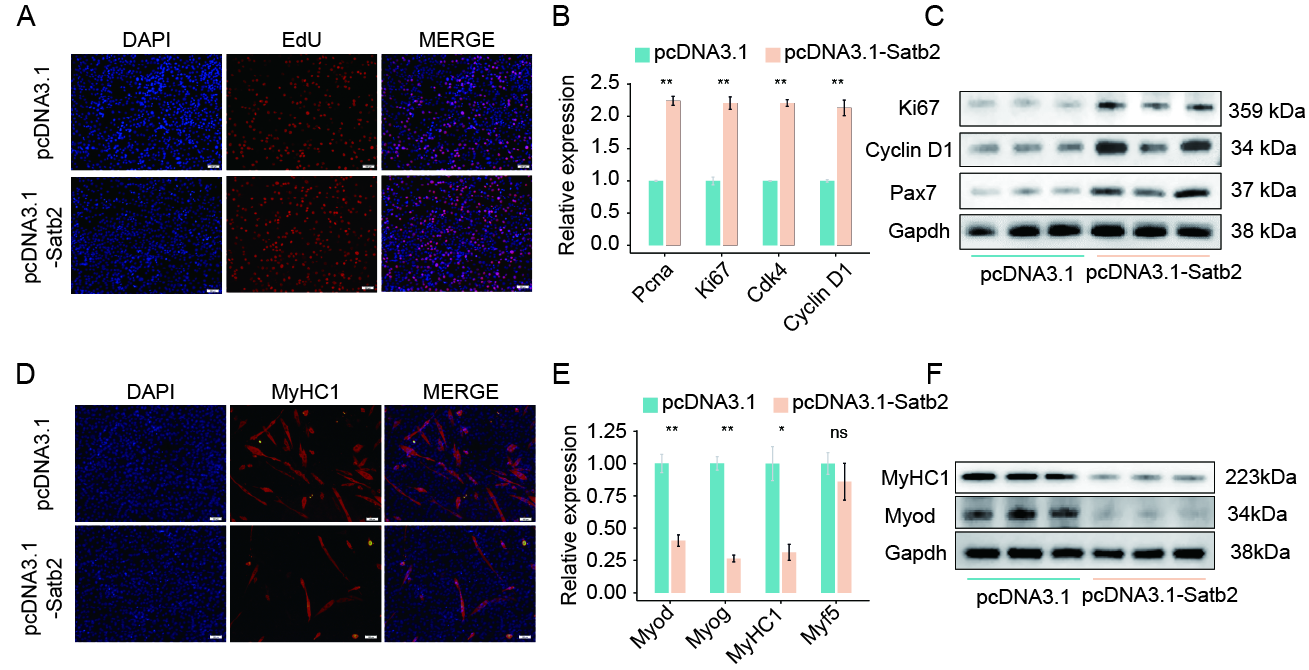

Supplement: S16 Fig — (A) EdU assay for proliferation of C2C12 myoblast cells following Satb2 overexpression. (B-C) qRT-PCR (B) and western blot (C) analysis of the expression levels of muscle proliferation markers following Satb2 overexpression in C2C12 cells. (D) Immunofluorescence assay for the differentiation of C2C12 myoblast cells following Satb2 overexpression. (E-F) qRT-PCR (E) and western blot (F) analysis of the expression levels of muscle differentiation markers following Satb2 overexpression in C2C12 cells. (TIF) [file pgen.1009910.s016.tif]
